# All‐Electrical Control of Spin Synapses for Neuromorphic Computing: Bridging Multi‐State Memory with Quantization for Efficient Neural Networks

**DOI:** 10.1002/advs.202417735

**Published:** 2025-04-26

**Authors:** Tzu‐Chuan Hsin, Chun‐Yi Lin, Po‐Chuan Wang, Chun Yang, Chi‐Feng Pai

**Affiliations:** ^1^ Department of Materials Science and Engineering National Taiwan University Taipei 10617 Taiwan

**Keywords:** field‐free switching, neuromorphic computing, neural network, perpendicular magnetic anisotropy, spin‐orbit torques

## Abstract

The development of energy‐efficient, brain‐inspired neuromorphic computing demands advanced memory devices capable of mimicking synaptic behavior to achieve high accuracy and adaptability. In this study, three types of all‐electrically controlled, field‐free spin synapse devices designed with unique spintronic structures presented: the Néel orange‐peel effect, interlayer Dzyaloshinskii‐Moriya interaction (i‐DMI), and tilted anisotropy. To systematically evaluate their neuromorphic potential, a benchmarking framework is introduced that characterizes cycle‐to‐cycle (CTC) variation, a critical factor for reliable synaptic weight updates. Among these designs, the tilted anisotropy device achieves an 11‐state memory with minimal CTC variation (2%), making it particularly suited for complex synaptic emulation. Through comprehensive benchmarking, this multi‐state device in convolutional neural networks (CNNs) using post‐training quantization is implemented. Results indicate that per‐channel quantization, particularly with the min‐max and mean squared error (MSE) observers, enhances classification accuracy on the CIFAR‐10 dataset, achieving up to 81.51% and 81.12% in ResNet‐18—values that closely approach the baseline accuracy. This evaluation underscores the potential of field‐free spintronic synapses in neuromorphic architectures, offering an area‐efficient solution that integrates multi‐state functionality with robust switching performance. The findings highlight the promise of these devices in advancing neuromorphic computing, contributing to energy‐efficient, high‐performance systems inspired by neural processes.

## Graphical abstract

This study develops three all‐electrically controlled, field‐free spintronic synapse devices for neuromorphic computing. The tilted anisotropy device achieves an 11‐state memory with minimal cycle‐to‐cycle variation (2%), enabling high‐accuracy neural network quantization (81.51% in ResNet‐18). These findings position spintronic synapses as a promising solution for energy‐efficient AI hardware.

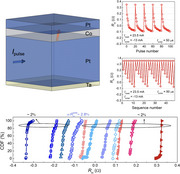

## 1 Introduction

The rapid rise of artificial intelligence (AI) has spurred demand for energy‐efficient and innovative computing solutions beyond traditional von Neumann architectures.^[^ ^1^, ^2^, ^3^, ^4^ ^]^ Neuromorphic computing, an unconventional computing paradigm that implements neural network algorithms on next‐generation hardware platforms, has gained significant attention.^[^ ^5^, ^6^, ^7^, ^8^ ^]^ Among various emerging non‐volatile memories, while resistive random‐access memory (RRAM) and phase‐change memory (PCM) offer high ON/OFF ratios and multilevel resistance states, their reliance on stochastic resistance tuning mechanisms, such as ion migration and phase transitions,^[^ ^9^, ^10^, ^11^ ^]^ causes material degradation and variability, limiting long‐term reliability.^[^ ^12^, ^13^, ^14^, ^15^, ^16^ ^]^ In contrast, spintronic synapses achieve deterministic multi‐state switching through domain wall motion, ensuring superior endurance, long retention times, and seamless CMOS compatibility.^[^ ^17^, ^18^ ^]^ By leveraging spin‐orbit torque (SOT), spintronics enables gradual domain nucleation or domain wall propagation, allowing multiple intermediate magnetization states that emulate synaptic weight updates and neural behavior.^[^ ^2^, ^19^, ^20^, ^21^, ^22^ ^]^ However, achieving precise electric control of domain wall motion remains a key challenge for their practical implementation. Additionally, SOT‐based memory devices often require an external magnetic field for deterministic switching due to symmetry constraints,^[^ ^23^, ^24^ ^]^ as seen in W/CoFeB/MgO‐based devices with perpendicular magnetic anisotropy (PMA)^[^ ^17^, ^25^ ^]^ and antiferromagnetic systems such as Pt/Co/NiO^[^ ^26^ ^]^ and Pt/Co/IrMn.^[^ ^27^ ^]^

Several studies have explored field‐free solutions to enhance the suitability of SOT devices for neuromorphic computing. For instance, the PtMn/[Co/Ni]_n_ system has demonstrated field‐free SOT switching via exchange bias, as shown by Kurenkov et al.^[^ ^8^, ^20^ ^]^ However, its current‐induced switching ratio is significantly lower than that of field‐driven switching, leading to substantial inefficiencies. Building on inversion symmetry characteristics, CoPt systems have exhibited analog‐like behavior under current pulses of varying amplitudes.^[^ ^19^, ^28^ ^]^ However, modulating memristive behavior through pulse amplitude rather than pulse number is impractical for hardware implementation, as it complicates circuit design and integration with peripheral systems.^[^ ^29^, ^30^ ^]^ Recently, FePt systems have shown promise for neuromorphic computing by incorporating field‐free mechanisms such as interlayer exchange coupling.^[^ ^31^, ^32^ ^]^ However, significant cycle‐to‐cycle (CTC) variations have been observed during memristive switching, particularly in the absence of an applied in‐plane field, compromising reliability and scalability for neuromorphic applications.^[^ ^33^ ^]^ Among existing field‐free methods, the CoPt system with tilted anisotropy stands out as a promising candidate. It not only enables pure current‐induced switching but also exhibits remarkably low CTC variation,^[^ ^34^, ^35^ ^]^ making it well‐suited for high‐precision synaptic weight updates. This necessitates a systematic study to elucidate the intricate relationship between field‐free SOT switching and stable analog‐like behavior.

In the realm of hardware implementation of neuromorphic computing using memristive devices, two key challenges have been widely recognized. First, while numerous studies have explored the use of discrete resistance states as weights in artificial neural networks (ANNs) with the MNIST dataset,^[^ ^19^, ^26^, ^31^, ^32^, ^36^ ^]^ research on their performance with more complex datasets, such as CIFAR‐10, and in more advanced architectures, such as convolutional neural networks (CNNs), remains limited.^[^ ^37^, ^38^, ^39^, ^40^ ^]^ Second, the quantization process, which is essential for bridging the gap between resistance states and software models, is often presented in a simplified manner or lacks comprehensive discussion. The most common approach relies on using the minimum and maximum values of the weight matrix during quantization,^[^ ^36^, ^41^, ^42^, ^43^, ^44^ ^]^ raising the question of whether this is the most effective method. Alternative techniques have been proposed in software‐based quantization range selection to mitigate potential errors,^[^ ^44^ ^]^ highlighting the need for further exploration of optimal quantization strategies.

Our study builds on this foundation by systematically addressing these gaps. In this work, we employ three types of optimized all‐electrical, field‐free spin synapse devices^[^ ^45^, ^46^ ^]^ to establish a benchmarking framework that spans different material designs and device structures, with the goal of achieving field‐free, ideal memristive behavior for neural network computation. Through pulse‐number experiments, each device is configured to enable distinct intermediate resistance states corresponding to multi‐level memory. This study introduces two key novelties. First, our systematic benchmarking approach allows for a direct comparison of synaptic performance across diverse device configurations. Second, we demonstrate an 11‐distinguishable‐state behavior in the tilted anisotropy device, identifying its low CTC variation and ideal characteristics for neural network computation. These resistance states are then integrated into convolutional neural networks (CNNs) through post‐training quantization (PTQ) with various observers, serving as a critical step in simulating hardware‐based inference. Specifically, PTQ approximates the mapping of floating‐point weights in neural network models to discrete resistance states in the devices, laying the groundwork for efficient hardware implementation. By bridging memristive spintronic devices with neural networks, this study provides a robust framework for synapse emulation in neuromorphic computing systems, advancing the potential for energy‐efficient and scalable artificial intelligence applications.

## 2 Results and Discussion

### 2.1 Field‐Free Current‐Induced Magnetization Switching

**Figure 1:**
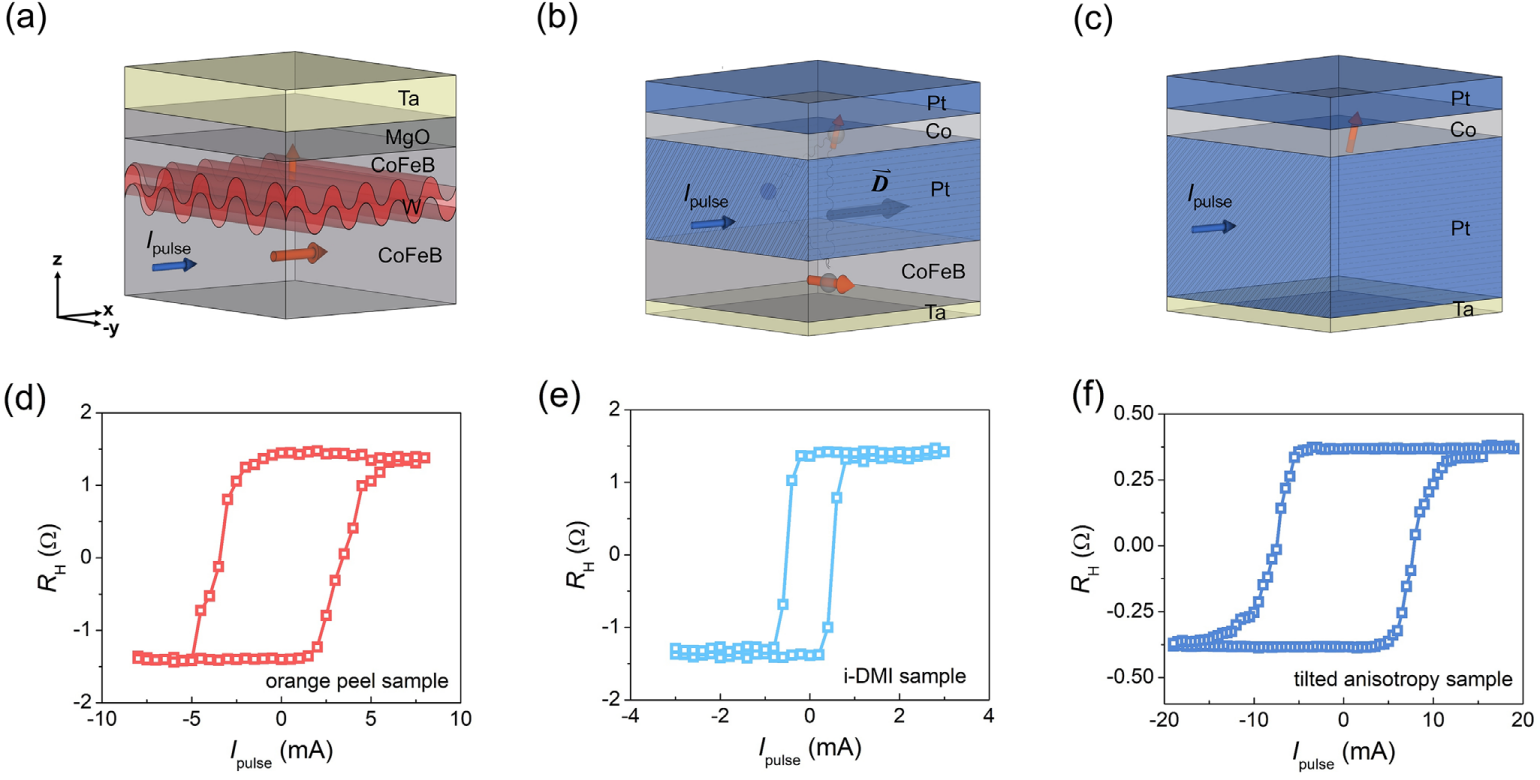
Three all‐electrical control devices with different field‐free solutions. Schematics of the samples with a) Sample I: the Néel orange‐peel effect, b) Sample II: the i‐DMI effect, and c) Sample III: tilted anisotropy. d–f) Representative current‐induced magnetization switching loops corresponding to the three field‐free solutions.

Three distinct stacked structures are prepared with different field‐free solutions to explore the all‐electric control spin synapse. **Figure** **1**a–c are the schematics of the different switching mechanisms. Figure 1d–f are the corresponding current‐induced magnetization switching results by gauging anomalous Hall resistance *R_H_* with a current pulse width of *t*_pulse_ = 50 ms.

Sample I: CoFeB(4)/W(1.4)/CoFeB(1.6)/MgO(1.1)/Ta(2), in which the field‐free current‐induced magnetization switching mechanism can be explained by the Néel orange‐peel effect^[^ ^46^, ^47^, ^48^, ^49^, ^50^, ^51^ ^]^ in Figure 1a,d. This effect originates from roughness‐induced magnetostatic coupling at the interfaces between the in‐plane and out‐of‐plane magnetic layers. Correlated surface roughness creates localized magnetic poles that give rise to a weak but stable in‐plane effective field, which serves as an intrinsic symmetry‐breaking field even in the absence of external magnetic bias.^[^ ^47^, ^50^ ^]^ A CoFeB(4)/W(1.4)/CoFeB(1.6) T‐type structure is utilized in this scenario, where in‐plane magnetic anisotropy (IMA) and PMA layers coexist. The red arrows represent the magnetization directions. With aligning the magnetization ***M*** of the in‐plane CoFeB layer along the current‐channel direction (***M***_CoFeB_ // ± *x*), the surface roughness of the CoFeB/W/CoFeB stack generates a significant Néel orange‐peel effective field within the structure, acting as a built‐in bias field (*H*_x_), which reorients the domain wall moments in the PMA layer to align parallel to ***M***_CoFeB_. This alignment facilitates domain wall propagation under the influence of the current‐induced SOT effective field.^[^ ^24^, ^52^ ^]^ However, the thickness of the W spacer layer is limited to 1.4 nm,^[^ ^46^, ^53^ ^]^ and a marked decline in performance is observed when the thickness increases to 2 nm, as indicated by Kao et al.^[^ ^46^ ^]^ Hence, SOT switching cannot be fully optimized, given that the 1.4 nm W layer is still smaller than its spin diffusion length (≈3 nm).^[^ ^54^, ^55^ ^]^ The corresponding zero‐field SOT effective fields and pulse width dependence of current‐induced magnetization switching are documented in Figure S1 (Supporting Information).

Sample II: Ta(0.5)/CoFeB(1.2)/Pt(2.5)/Co(0.6)/Pt(0.6)/Ta(2), which possesses the strong i‐DMI effect^[^ ^45^, ^56^, ^57^, ^58^, ^59^, ^60^ ^]^ as shown in Figure 1b,e. The i‐DMI originates from spin‐orbit coupling in systems with broken inversion symmetry, particularly in ferromagnet/heavy metal/ferromagnet heterostructures.^[^ ^56^, ^58^ ^]^ Herein, CoFeB(1.2) has IMA while Co(0.6) sandwiched between two Pt layers exhibits PMA, building a T‐type structure. However, as indicated by the red arrows, the magnetization of the in‐plane CoFeB layer aligns perpendicularly with the current channel (***M*** // ±*y*). The coherent switching between the in‐plane CoFeB and the PMA Co layers makes the current‐induced switching possible via the DMI energy form ‐***D*** ∙(***M***_Co_× ***M***_CoFeB_)^[^ ^45^, ^58^, ^60^ ^]^ with the oblique deposition of the spacer Pt. The wave function interference at both Pt/Co interfaces leads to oscillatory interlayer exchange coupling, where the strength of the i‐DMI depends sensitively on the capping thickness. The optimized asymmetric Pt design enhances the i‐DMI strength through constructive interference of the electron wave functions.^[^ ^60^ ^]^ This strong coupling leads to efficient current‐induced magnetization switching with a large current‐induced effective field *H*_z_^eff^/*I*_DC_ of 9.5 Oe mA^−1^ and 100% switching ratio (current‐scan vs field‐scan), as detailed in Figure S2 (Supporting Information). Please note that the orientation of the ***D***‐vector should align along the *x‐*axis.

Sample III: Ta(0.5)/Pt(6.1)/Co(0.7)/Pt(1), which is the tilted anisotropy sample^[^ ^24^, ^45^, ^61^, ^62^, ^63^ ^]^ in Figure 1c,f. As implied by the name, the anisotropy axis in this device is deliberately tilted away from the *z*‐axis. This is achieved by obliquely depositing the bottom Pt layer. The tilted anisotropy originates from the structural modifications in the Pt(6) layer, where the (111) texture tilts due to the deposition angle. This tilt affects the neighboring Co layer, leading to ***M***_Co_ (the red arrow) tilt from the *z*‐axis toward the *y*‐*z* plane.^[^ ^63^ ^]^ One classical trend of the tilted anisotropy device is the nonlinear trend of the current‐induced effective field, which has been verified experimentally.^[^ ^45^, ^63^ ^]^ This increasing *H*_z_^eff^ with the applied current pulse also leads to a 100% field‐free current‐induced SOT switching, as shown in Figure S3 (Supporting Information). The objective of this study is to compare and identify the most effective field‐free switching mechanism for neuromorphic computing applications, as detailed in the subsequent experimental section.

### 2.2 Nonvolatile 11‐State Memory

**Figure 2:**
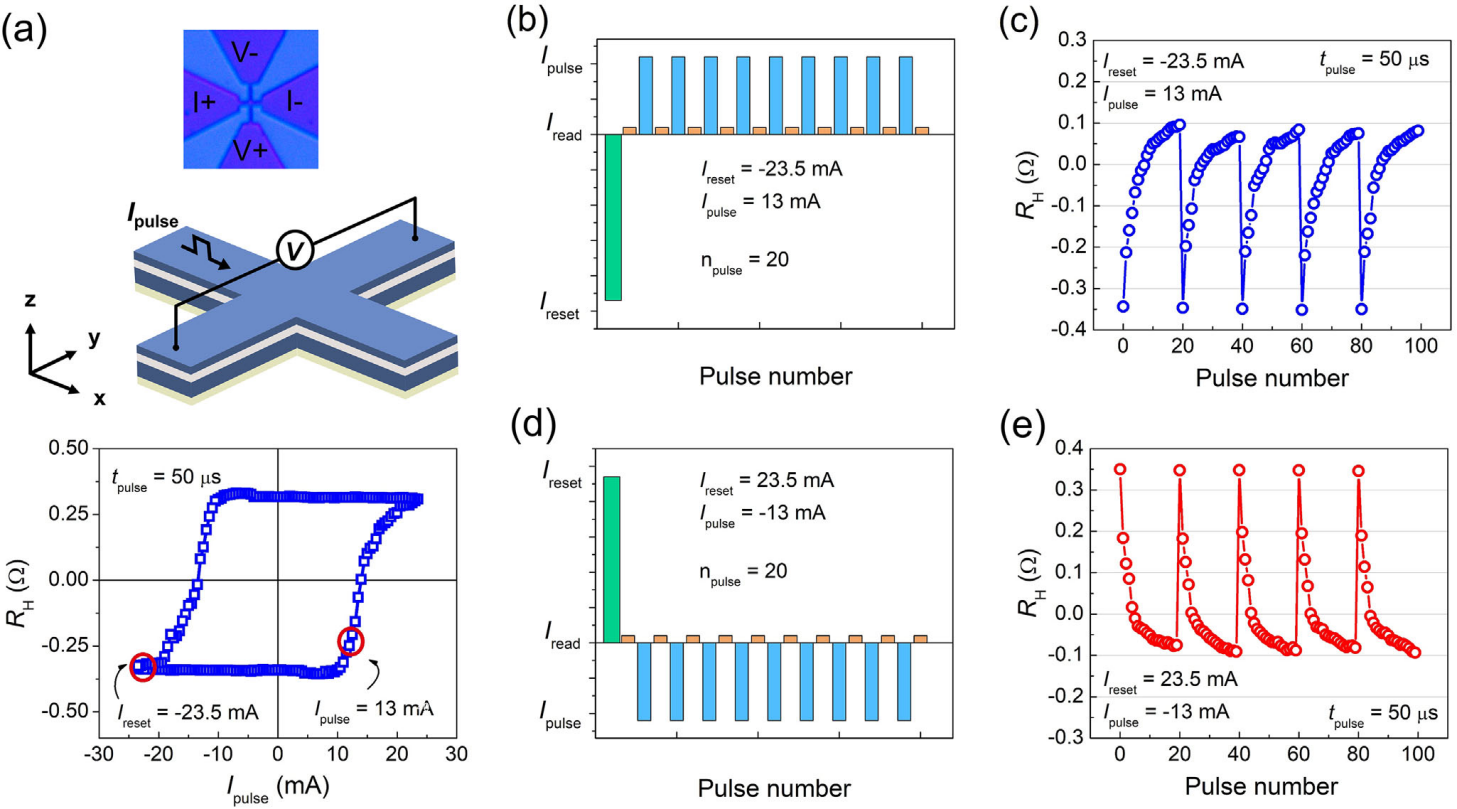
Memristive behaviors in the tilted anisotropy device (Sample III) with sequential 50 µs current pulses. a) The upper panel shows the Hall bar device and coordinate system for electrical measurements. The lower panel demonstrates current‐induced magnetization switching under a current pulse of 50 µs. b) The first applied train of current pulses and c) the corresponding response of *R_H_*, where equal 19 positive current pulses of 13 mA are applied after a large reset current of −23.5 mA. d) The second applied train of current pulses and e) the corresponding response of *R_H_*, where equal 19 positive current pulses of −13 mA are applied after a large reset current of 23.5 mA.

Extensive electrical measurements are conducted to evaluate the memristor's ability to store multiple states and emulate synaptic behavior. **Figure** **2**a illustrates the transport measurement setup and the detection of current‐induced magnetization switching via changes in *R_H_* ​, with *t*_pulse_​ set to 50 µs in the tilted anisotropy device (Sample III). This duration represents the minimum time required for complete switching in the micron‐size sample, as shown in Figure S3 (Supporting Information). Furthermore, the selection of a 50 µs pulse width and a pulse amplitude of 13 mA are determined through systematic optimization to maximize the number of distinguishable resistance states while minimizing the CTC variation (details provided in Section S4, Supporting Information). The sequence of current pulses is illustrated in Figure 2b. The process begins by aligning the magnetization downward (‐*z*‐direction) using a large reset current (*I*_reset_) of −23.5 mA. Subsequently, 19 consecutive current pulses (*I*_pulse_), each with *t*_pulse_ = 50 µs and a consistent magnitude of 13 mA, are applied. Read current pulses are then applied after each *I*_reset_ and/or *I*_pulse_, with a magnitude of 0.1 mA and a pulse width of 50 ms. The resulting *R_H_* readout, shown in Figure 2c, exhibits a gradual increase characteristic of long‐term potentiation (LTP), eventually reaching a saturated state with nonlinear behavior.^[^ ^64^ ^]^ Moreover, Figure 2c reveals that the Hall resistance difference ∆*R_H_*, when switched by pulses of uniform magnitude, is ≈68% of the ∆*R_H_* observed in Figure 2a, where pulses of varying amplitude are used. Notably, each cycle exhibits remarkable reproducibility. Following the same procedure, the process can be conducted in reverse, as shown in Figure 2d, where *I*_reset_ = 23.5 mA and the *I*_pulse_ = −13 mA. The resulting *R_H_* readout, shown in Figure 2e, exhibits a gradual decrease, indicative of long‐term depression (LTD). Hence, the full range of magnetization states can be accessed by applying two opposing trains of current pulses.

**Figure 3:**
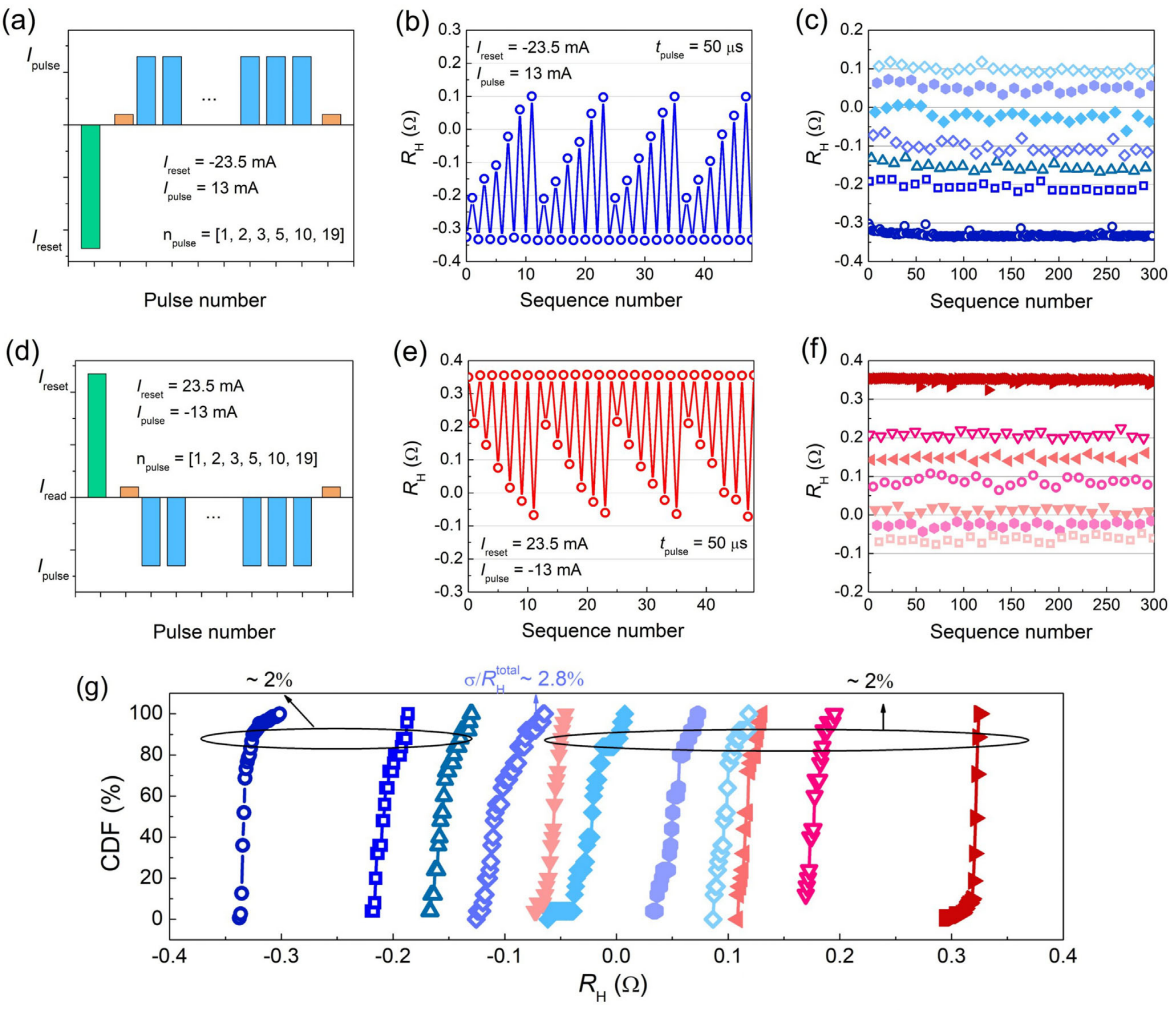
Demonstration of 11 distinguishable states in the tilted anisotropy device (Sample III) with sequential 50 µs current pulses. a,d) The applied train of six specific current pulse numbers (coined as sequences), and b,e) the corresponding response of *R_H_*. Seven distinguishable states were obtained by c) the first applied chain (down‐to‐up) and f) the second applied chain (up‐to‐down). g) The cumulative distribution functions (CDFs) versus *R_H_* for 11 distinguishable states.

A further set of measurements is conducted to determine the number of stable states and assess the reliability and range of resistance variation. As shown in **Figure** **3**a, a new pulse sequence is applied using the same *I*_reset_ and *I*_pulse_ values. Read currents are introduced after a set number of pulses at specified intervals (n_pulse_ = 1, 2, 3, 5, 10, 19), with these intervals increasing as *R_H_​* approaches saturation to ensure that the multilevel resistance states are evenly distributed. The read current is applied only after *I*_reset_ and at these six specified pulse intervals. Figure 3b reveals seven distinct resistance states that emerge in response to repeated sequences of these seven pulse numbers. To quantify the variability of each state, 25 data points are collected for each of the seven distinct magnetic states by repeating the sequence accordingly, as shown in Figure 3c. Similarly, by reversing the pulse sequence (*I*_reset_ = 23.5 mA and *I*_pulse_ = −13 mA), the opposite resistance modulation could be achieved, as demonstrated in Figure 3d,e. Another set of seven distinct states was collected, as illustrated in Figure 3f.

With these two pulse sequences, the full range of *R_H_* values is expressed through a total of 11 discrete states, eliminating overlapping states. To quantify the CTC variability, the standard deviation (σ) of the 25 data points per state is computed, and cumulative distribution functions (CDFs) are constructed against the measured *R_H_* ​ to visualize this variation, as shown in Figure 3g.^[^ ^33^ ^]^ To provide a quantitative and comparable benchmark for evaluating material and device design, the CTC variation is defined as the ratio σ/$R_{H}^{\mathrm{total}}$, where $R_{H}^{\mathrm{total}}$ represents the total Hall resistance difference switched by current pulses. The highest CTC variation among the 11 states is 2.8%, while the rest do not exceed 2%, confirming the robustness and stability of the 11 discrete resistance states in the tilted anisotropy device. Furthermore, the same multilevel switching behavior is reproduced across three devices fabricated under identical conditions, each exhibiting at least 9 distinguishable states with CTC variations below 2.7% (Figure S10, Supporting Information), further validating the consistency and robustness of our approach.

In addition to demonstrating the capability of achieving 11 distinguishable resistance states, it is crucial to highlight the advantage of the tilted anisotropy device in minimizing CTC variation compared to other emerging memory technologies. The average CTC variation of 2% surpasses that of RRAM and PCM, which typically exhibit variations ranging from 10% to 100%.^[^ ^12^, ^13^, ^14^, ^15^, ^33^ ^]^ This discrepancy can be attributed to the stochastic resistance tuning mechanisms in RRAM and PCM, which stem from ion migration and phase transition effects,^[^ ^9^, ^10^, ^11^ ^]^ introducing uncertainty in neuromorphic computing. In contrast, the stable multilevel states in our device underscore the feasibility of spintronic synapses as a robust and energy‐efficient alternative.

In this study, we utilize *R_H_* ​ from the anomalous Hall effect (AHE) as the readout parameter to verify the physical feasibility of achieving and controlling 11 discrete resistance states, which forms the foundation for our subsequent simulation and application discussions. Our results demonstrate that through precise domain wall motion control, stable intermediate states can be achieved without external magnetic fields, with a CTC variation of only ≈2%, confirming the robustness and reproducibility of this multi‐state behavior. This represents a significant advancement compared to previous works,^[^ ^8^, ^17^, ^19^, ^20^, ^25^, ^26^, ^27^, ^28^ ^]^ where CTC variations were rarely quantified, leading to unreliable resistance states for neuromorphic computing applications. While *R_H_* measurements effectively validate multi‐state switching, practical large‐scale implementations may require alternative readout mechanisms with higher signal contrast and lower variability. One promising approach is tunnel magnetoresistance (TMR) in magnetic tunnel junctions (MTJs), which can provide a significantly higher ON/OFF ratio, typically exceeding several hundred percent.^[^ ^65^ ^]^ Such an enhancement would improve state distinguishability and enable more precise weight updates in neuromorphic architectures. Although *R_H_* is sufficient for fundamental verification, integrating an MTJ‐based readout could further enhance robustness, making spintronic synapses more viable for large‐scale applications.^[^ ^29^, ^30^ ^]^

### 2.3 The Benchmark of Three Field‐Free Solutions for Neuromorphic Computing

In analyzing the suitability of three distinct field‐free spin synapse devices for neuromorphic computing applications, their fundamental switching characteristics and memristive behaviors are examined. Identical pulse number measurements are performed on the Néel orange‐peel effect and i‐DMI samples to determine σ/$R_{H}^{\mathrm{total}}$ and the available stable states (see Figures S7 and S8 in the Supporting Information) with **Table** **1** summarizing the comparative benchmarks.

**Table 1:** The benchmark of three field‐free solutions for neuromorphic computing.

	Sample I, Néel orange‐peel effect	Sample II, i‐DMI	Sample III, Tilted anisotropy
Switching ratio (%) current‐scan vs field‐scan	71	100	100
Switching ratio (%) pulse number vs field‐scan	34	100	68
Effective field (*H*_z_^eff^/*I*_DC_)	3.4	9.7	3.5
*J*_pulse_ (10^10^ A/m^2^)	9 @50ms	17 @10µs	32 @50µs
CTC variation (σ/$R_{H}^{\mathrm{total}}$, %)	7.2	4.5	2.8
Available state number	3	4	11
Thermal stability factor	56.5	20.8	30

First, evaluating the switching performance is crucial for the memristive window, comparing the switching ratio driven by the field scan, the current scan, and the pulse number metrics. For example, in the Néel orange‐peel effect sample, current‐induced switching yields a switching ratio of 71% during field scans, whereas the pulse number experiment shows a reduced ratio of 34%. This reduction can be attributed to the relatively modest current‐induced effective field ($H_{z}^{\mathrm{eff}}$/*I*_DC_ = 3.4 Oe mA^−1^), which is likely a consequence of the thin 1.4 nm W layer. This layer represents a trade‐off between enhancing the Néel orange‐peel effect and maintaining an optimal SOT performance.^[^ ^46^ ^]^ In contrast, the i‐DMI sample maintains a 100% switching ratio across different tests, thanks to its robust current‐induced effective field of $H_{z}^{\mathrm{eff}}$/*I*_DC_ = 9.7 Oe mA^−1^. This large value is generated by the coherent switching of the magnetic layers within the strong i‐DMI coupling, which is induced by the asymmetric Pt configuration on both sides of the Co layer and the resulting electron wave function interference.^[^ ^45^, ^60^ ^]^ For the tilted anisotropy sample, the current‐induced effective field increases with the applied current, enabling a 100% switching ratio in the current scan mode. This characteristic can be attributed to a spin‐transfer‐torque‐like behavior, and the nonlinear trend in the current‐induced effective field, as shown in Figure S3c (Supporting Information), has also been reported in previous works.^[^ ^45^, ^63^ ^]^ Nevertheless, a non‐saturated 68% is achieved due to the specific amplitude pulse applied to generate the same effective field. The pulse width selection follows the minimum duration required for full switching in each sample.^[^ ^20^ ^]^ Notably, the Néel orange‐peel effect sample requires a pulse width of 50 ms due to the high resistivity of its W/CoFeB‐based heterostructure. This requirement highlights the advantages of Pt‐based devices, which offer lower resistivity and higher energy efficiency.

Beyond switching performance, the number of intermediate states available in each device varies significantly. The Néel orange‐peel effect sample exhibits only three intermediate states, as indicated by its large σ/$R_{H}^{\mathrm{total}}$​ value of 7.2% and a relatively low switching ratio. The i‐DMI sample, despite its moderate σ/$R_{H}^{\mathrm{total}}$​ value of 4.5% and 100% switching ratio, also supports a limited number of four intermediate states. The strong current‐induced effective field in this sample favors a binary switching mechanism, as shown in Figure S8b (Supporting Information).

Thermal stability is another crucial parameter for evaluating device performance, particularly in field‐free spintronic applications. The Néel orange‐peel effect sample exhibits a high thermal stability factor of ∆ = 56.5, making it suitable for storage memory applications that require long retention times exceeding ten years. The i‐DMI sample, in contrast, offers robust current‐induced switching with low power consumption, characterized by a zero thermal critical switching current density of *J*_c0_ ≈ 2.05  ×  10^11^ A m^−2^ and distinct binary behavior. These characteristics position it as an ideal candidate for last‐level cache memory applications, where a lower thermal stability factor of 20.8 is permissible due to the frequent refreshing of data.^[^ ^66^ ^]^ Ultimately, the tilted anisotropy sample is the most promising candidate for neuromorphic computing applications due to its moderate current‐induced effective field, a thermal stability factor of ∆ ≈ 30, and the presence of 11 intermediate states, which enable complex synaptic function emulation. Note that in this benchmark, device‐to‐device variation is carefully evaluated. As discussed in Section 2.2 and illustrated in Figures S9 and S10 (Supporting Information), the tilted anisotropy devices consistently demonstrated robust multilevel switching behavior across samples, confirming minimal impact from fabrication‐induced variations.

### 2.4 Implementation of 11 Distinguishable States to the Convolutional Neural Network

#### 2.4.1 Artificial Weights Quantization

**Figure 4:**
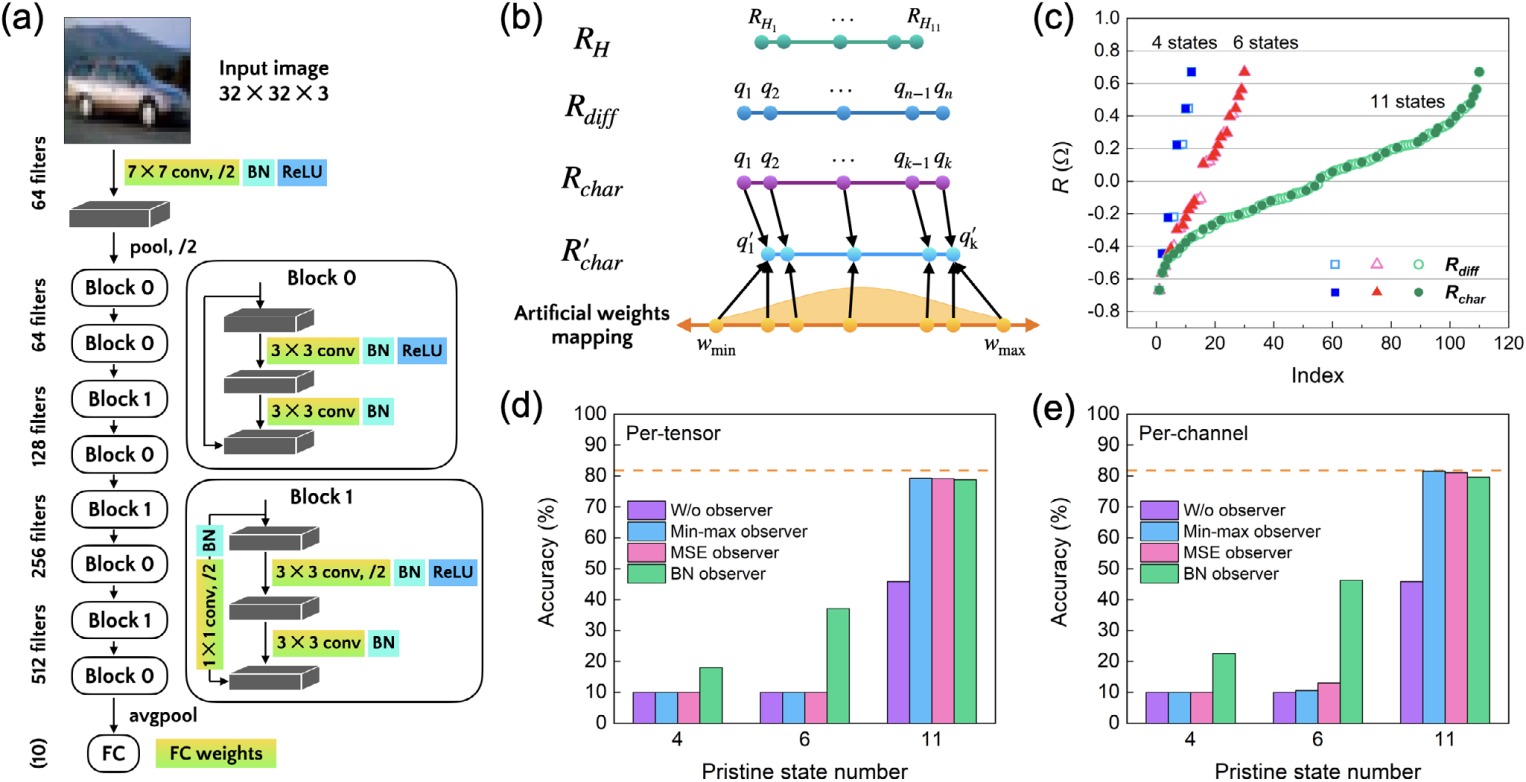
Application of artificial weights in the CNN model using 11 distinguishable states. a) Architecture of ResNet‐18, including max‐pooling layers, batch normalization (BN), ReLU activation, fully connected (FC) layers, and convolution layers. The blocks with a yellow‐green gradient represent modules for weight quantization. b) Illustration of the PTQ process, showing the conversion of pristine Hall resistance states (***R***_***H***_) into differential resistance states (***R***_***diff***_), the extraction of characteristic resistance states (***R***_***char***_), and ***R***_***char***_ after range adjustment ($R_{\mathrm{char}}^{\prime }$). (c) Dependence of ***R***_***char***_ and ***R***_***diff***_ on the state index for 4, 6, and 11 pristine states. Classification accuracy of the per‐tensor d) and per‐channel e) quantized ResNet‐18 model with 4, 6, and 11 pristine states, with and without observers. The dashed line represents the baseline accuracy of 81.78% for ResNet‐18.

To demonstrate the capability of our multi‐state device in neuromorphic computing, the 11 distinguishable states from the tilted anisotropy sample are applied as artificial weights to a representative convolutional neural network, ResNet‐18. Using the PyTorch framework, the architecture of ResNet‐18, shown in **Figure** **4**a, includes pooling layers, batch normalization (BN), ReLU activation, fully connected (FC) layers, and convolutional layers. The 18 weight layers in ResNet‐18 comprise 17 convolutional layers and one FC layer, where convolutional layers store weight tensors of size ***C***_***in***_× ***K*** × ***K*** × ***C***_***out***_, with ***C***_***in***_ and ***C***_***out***_ representing the number of input and output channels, respectively, and ***K*** denoting the kernel size. Leveraging a residual learning framework, ResNet‐18 excels in image recognition, achieving training and testing accuracies of 88.07% and 81.78%, respectively.

The simulated on‐chip inference of ResNet‐18 is conducted through a PTQ process, where software‐based weights are mapped to quantized resistance states of the devices, as illustrated in Figure 4b. The pristine anomalous Hall resistance states (***R***_***H***_) are first converted into differential resistance states (***R***_***diff***_) by calculating the resistance difference between pairs of memristors. This step is critical for generating negative resistance (or conductance), which cannot be directly represented by a single memristor.^[^ ^41^, ^67^, ^68^ ^]^ The resulting ***R***_***diff***_ values, spanning the full range from negative to positive (*q*_1_ to *q_n_*), are thus generated. However, since ***R***_***H***_​ in our experiment is not evenly spaced, the intervals between resistance states may be too narrow to differentiate effectively, particularly when accounting for the device's inherent variability, such as nonlinearity and asymmetric resistance modulation.^[^ ^64^, ^69^ ^]^ To address these non‐ideal characteristics, several measures are implemented. Representative resistance values are chosen by ensuring the difference between any two ***R***_***diff***_ values meets a minimum threshold, defined as a certain percentage of the total resistance range. In this study, a pre‐examined 2% threshold is applied:

(1) $$q_{k}-q_{k-1}\geq 0.02\;\left(q_{n}-q_{1}\right)$$

This process produces characteristic resistance states (***R***_***char***_), resulting in 36 weight levels (*q*_1_ to *q_k_*) derived from 11 pristine states. Similarly, ***R***_***char***_ is calculated for 4 and 6 out of the 11 pristine states, yielding 6 and 22 weight levels, respectively (details are provided in Section S8, Supporting Information). The dependence of ***R***_***char***_ and ***R***_***diff***_ on the state index for 4, 6, and 11 pristine states is shown in Figure 4c. Note that the distribution of weight levels becomes increasingly uneven as the number of pristine states decreases.

Once ***R***_***char***_ is determined, the software weights can be preliminarily mapped. However, the quantization accuracy (∼10‐45%) often deviates significantly from the baseline, particularly in cases involving lower weight levels. This discrepancy arises because ***R***_***char***_​ lacks values near zero, leading to substantial quantization errors for values that are primarily distributed around the mean value of zero in the ResNet‐18 model, as illustrated in Figure S10 (Supporting Information).

To address this issue and bridge the gap between the software and hardware weights, a refined set of characteristic states denoted as $R_{\mathrm{char}}^{\prime }$, can be obtained by applying a quantization range adjustment to ***R***_***char***_​ using a linear transformation:^[^ ^44^, ^70^ ^]^

(2)

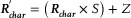

where the scaling (*S*) and offset (*Z*) coefficients are determined as follows:

(3) $$S=\frac{q_{max}-q_{min}}{q_{k}-q_{1}}\;\mathrm{and}\;Z=\left(q_{k}\times S\right)-q_{max}\;$$

The minimum (*q_min_*) and maximum (*q_max_*) weights can be derived using various observers, such as min‐max, mean square error, and batch normalization, which will be discussed in detail.

First, in the min‐max observer, for any given weight matrix ***W***, the *q_min_* and *q_max_* values are determined as follows:

(4) $$q_{min}=\min\left(W\right)\;$$

(5) $$q_{max}=\max\left(W\right)\;$$

This approach ensures that the full dynamic range of the original 32‐bit floating‐point (FP32) weight matrix is preserved during quantization. Additional details on the quantization of specific weight layers after applying the min‐max observer are presented in Figure S10 (Supporting Information).

Despite the widespread use of the min‐max observer for defining *q_min_* and *q_max_*, this method has a significant drawback, which is its sensitivity to large outliers in the weight matrix. Given the limited number of states in our device, ensuring that no clipping errors occur may result in excessive rounding errors. Therefore, it is essential to explore alternative quantization observers and assess their performance across different quantization scenarios. The mean squared error (MSE) method addresses this issue by determining. *q_min_* and *q_max_* as follows:^[^ ^44^ ^]^

(6) $$\underset{q_{\min},q_{\max}}{\arg\;\min}{\left\|W-\hat{W}\left(q_{\min},q_{\max}\right)\right\|}_{F}^{2}$$

where $\hat{W}\left(q_{min},\;q_{max}\right)$ represents the quantized version of ***W***, and ‖ · ‖_*F*_ denotes the Forbenius norm. Minimizing the MSE between ***W*** and $\hat{W}$ provides an effective approach for introducing clipping errors in a controlled manner.

The third observer employed in this study, the batch normalization (BN) observer, determines *q_min_* and *q_max_* as follows:

(7) $$q_{min}=\mu -N\times \sigma$$

(8) $$q_{max}=\mu +N\times \sigma$$

where $N\in R^{+}$, and *μ* and *σ* are the mean and standard deviation of the weight matrix ***W***, respectively. By selectively defining *q_min_* and *q_max_* for specific weight tensors using an optimized *N* value, the BN observer effectively mitigates the impact of large outliers. Further details on this method can be found in Section S9 (Supporting Information).

In our study, we focus on applying discrete states to neural network weights. While prior work has explored simulating activation functions via memristive switching loops,^[^ ^19^, ^26^, ^32^ ^]^ experimental validation of their role in vector‐matrix multiplication (VMM) or multiply‐accumulate (MAC) operations remains lacking.^[^ ^42^, ^67^, ^71^ ^]^ Recent pioneering studies have investigated the prototype of neuron circuit design, but further research is needed to establish its feasibility.^[^ ^72^ ^]^ Moreover, software and hardware weights can often be considered interchangeable, as the original values can be recovered by applying the inverse of the scaling factor.^[^ ^41^, ^64^ ^]^ This makes PTQ a viable approach for hardware weight mapping. In our work, we implement a memristor‐based CNN by employing a linear transformation to align hardware weights with the corresponding software weight range. This method is highly adaptable and can be extended to both simpler and more complex neural network architectures. For instance, a demonstration of the PTQ process in a multilayer perceptron (MLP) model is provided in Section S10 (Supporting Information).

#### 2.4.2 Per‐Tensor Quantization

Given the complexity of 4‐D tensors, convolutional weights can be quantized using either per‐tensor or per‐channel schemes. In the per‐tensor quantization approach, quantization parameters are applied to the entire weight tensor of a layer, treating it as a single unit. This method is similar to the quantization strategy used for fully connected layers. Detailed weight statistics before and after quantization are presented in Figure S13 (Supporting Information). After applying these quantization methods, the overall quantization accuracy generally improves compared to cases without an observer, as shown in Figure 4d. Among the methods, the min‐max and MSE observers are particularly effective for CNN per‐tensor quantization, achieving accuracies close to the baseline at 79.22% and 79.12%, respectively. Furthermore, the dependence of quantization accuracy on the number of states is analyzed, demonstrating that only when using artificial weights with 11 pristine states does the accuracy approach the baseline. This finding highlights the importance of using a higher number of artificial weights for achieving optimal performance in hardware‐implemented neural networks.

#### 2.4.3 Per‐Channel Quantization

While per‐tensor quantization is suitable for many cases, its accuracy is relatively low when the number of pristine states is as low as 4 or 6, and even with 11 pristine states, there is still room for improvement. To overcome this limitation, per‐channel quantization is employed, where quantization parameters are defined independently for each segment of output channels within a layer.^[^ ^44^, ^73^ ^]^ A detailed analysis of weight transfer error distributions for the first convolutional layer across all output channels is presented in Figure S14 (Supporting Information). Per‐channel quantization consistently demonstrates higher accuracy compared to per‐tensor quantization across all weight levels and for all three observers in the ResNet‐18 model, as shown in Figure 4e. Notably, the BN observer achieves improved accuracy with only six states, while the highest quantization accuracy is obtained with 11 states—reaching 81.51% and 81.12% for the min‐max and MSE observers, respectively—which is close to the baseline performance. A detailed comparison of classification accuracies for various quantization schemes in ResNet‐18 is provided in Table S1 of the Supporting Information.

## 3 Conclusion

This study demonstrates the potential of all‐electrical, field‐free spin synapse devices for neuromorphic computing applications. Through a systematic exploration of three distinct spintronic structures, including the Néel orange‐peel effect, i‐DMI, and tilted anisotropy, we identify these configurations as promising candidates for achieving stable and efficient multi‐state magnetization switching. Our findings emphasize that the tilted anisotropy device, with its robust 11‐state memory capability, moderate thermal stability (∆ ≈ 30), and an average CTC variation of ≈2%, excels in simulating complex synaptic functions, making it particularly suitable for advanced neuromorphic computing systems. Meanwhile, the Néel orange‐peel and i‐DMI structures offer advantageous trade‐offs in endurance and switching efficiency, providing versatility for different hierarchical memory levels within neuromorphic architectures.

Furthermore, we validate the application of these multistate devices in ResNet‐18 through PTQ processes, where adjusting the quantization range via linear transformation is crucial to managing the nonlinear LTP and LTD resistances. By mapping device resistance states to quantized weight levels, we achieve reliable weight transformation for CNN models, demonstrating high classification accuracy with minimal quantization error. The BN observer exhibits strong quantization performance even with fewer weight levels, highlighting its potential for efficient quantization in constrained settings. Notably, per‐channel quantization with the MSE (min‐max) observer achieves an impressive accuracy of 81.51% (81.12%), surpassing per‐tensor quantization and demonstrating its effectiveness in capturing finer weight variations in CNNs. This result underscores the flexibility of these spintronic devices in supporting diverse quantization strategies for real‐world AI applications. Future work includes investigating on‐chip learning algorithms^[^ ^39^, ^40^, ^74^, ^75^ ^]^ and linearly discretized resistance modulation in spintronic devices through tilted anisotropy, with potential applications in spiking neural networks^[^ ^35^, ^76^, ^77^ ^]^ and image edge detection.^[^ ^28^ ^]^

## 4 Experimental Section

### Sample Growth

Three distinct stacked structures are prepared by magnetron sputtering: Sample I: CoFeB(4)/W(1.4)/CoFeB(1.6)/MgO(1.1)/Ta(2), in which the current‐induced magnetization switching can be explained by the Néel orange‐peel effect. Sample II: Ta(0.5)/CoFeB(1.2)/Pt(2.5)/Co(0.6)/Pt(0.6)/Ta(2), which possesses the strong i‐DMI effect. Sample III: Ta(0.5)/Pt(6.1)/Co(0.7)/Pt(1), the tilted anisotropy sample. Sample I is sputtered with the sample holder rotated at 10 revolutions per minute (RPM). Sample II and Sample III are sputtered with the bottom Pt layer (2.5 and 6.1 nm Pt respectively) being obliquely deposited without rotating the sample holder, while the remaining layers are prepared with a rotating speed of 10 RPM. All three samples are deposited onto SiO_2_ substrates in an ultra‐high vacuum magnetron sputtering system with a base pressure of 5  ×  10^−8^ Torr. The metallic (oxide) layers are deposited by DC (RF) sputtering under an Ar growth pressure of 3 mTorr. The composition of CoFeB is 20%, 60%, and 20%, respectively. The numbers in the parenthesis are the thickness of each layer in nanometers. The films are deposited via DC magnetron sputtering at room temperature and an Ar growth pressure of 3 mTorr. The bottom Ta serves as a seed layer to ensure the uniformity of the devices, and Ta(2) layers are used to cap the stacks and provide protection.

### Device Fabrication

The samples are patterned into Hall bar devices with a current channel width of 5 µm and voltage arm width of 3 µm through photolithography and lift‐off process.

### Electrical and Magnetic Measurement

*R_H_* is measured by a homemade probe station, which has a projected vector field magnet capable of simultaneously applying in‐plane and out‐of‐plane magnetic fields. Electrical measurements are performed with a DC source (by Keithley 2400) and a voltage meter (by Keithley 2000). For the pulse number measurement, the write current pulses shorter than 10 ms are generated by an Agilent Pulse Generator 81110A.

### Dataset and Training Conditions

The CNN simulation utilizes the CIFAR‐10 dataset, consisting of 40 000 training images, 10 000 validation images, and 10 000 testing images, each with dimensions of 32 × 32 × 3. ResNet‐18 is trained using pre‐trained weights with a mini‐batch size of 32, a learning rate of 0.001, and over five epochs.

## Conflict of Interest

The authors declare no competing interests.

## Supporting information



Supporting Information

## Data Availability

The data that support the findings of this study are available from the corresponding author upon reasonable request.
