# Supplementary material for: All‐Electrical Control of Spin Synapses for Neuromorphic Computing: Bridging Multi‐State Memory with Quantization for Efficient Neural Networks
Source: Adv Sci (Weinh). 2025 Apr 26;12(22):2417735. doi: 10.1002/advs.202417735 (PMC12165024; doi:10.1002/advs.202417735)
Supplement: Supplementary file 1 — Supporting Information [file ADVS-12-2417735-s001.pdf]

ADVANCED SCIENCE
Open Access

Supporting Information

for *Adv. Sci.*, DOI 10.1002/advs.202417735

All-Electrical Control of Spin Synapses for Neuromorphic Computing: Bridging Multi-State Memory with Quantization for Efficient Neural Networks

*Tzu-Chuan Hsin, Chun-Yi Lin, Po-Chuan Wang, Chun Yang and Chi-Feng Pai**

Supporting Information

All-Electrical Control of Spin Synapses for Neuromorphic Computing: Bridging Multi-State Memory with Quantization for Efficient Neural Networks

Tzu-Chuan Hsin[1†], Chun-Yi Lin[1†], Po-Chuan Wang[1], Chun Yang[1], Chi-Feng Pai[1]*

T.-C. Hsin, C.-Y. Lin, P.-C. Wang[+], C. Yang, C.-F. Pai

Department of Materials Science and Engineering, National Taiwan University

Taipei 10617, Taiwan

*E-mail: cfpai@ntu.edu.tw

[+]Present address: School of Electrical and Computer Engineering, Georgia Institute of Technology, Atlanta, GA 30332, USA.

Table of Contents

S1. Field-free SOT switching with Néel orange-peel effect

S2. Field-free SOT switching with i-DMI

S3. Field-free SOT switching with tilted anisotropy

S4. Optimization of pulse width and current amplitude for multi-state switching

S5. Distinguishable states in the Néel orange-peel effect device

S6. Distinguishable states in the i-DMI device

S7. Assessment of device-to-device variations

S8. The characteristic resistance states (R_{char}) with various pristine states

S9. The optimization of accuracy using the batch normalization (BN) observer

S10. Application of post-training quantization process in a multilayer perceptron model

S11. Weight statistics for per-tensor quantization

S12. Weight statistics for per-channel quantization

S13. Comparison of classification accuracy for three quantization schemes in CNN model

---

† Tzu-Chuan Hsin and Chun-Yi Lin contributed equally to this work.

S1. Field-free SOT switching with Néel orange-peel effect

Figure S1(a) is the display the out-of-plane (OOP) hysteresis loop of Sample I: CoFeB(4)/W(1.4)/CoFeB(1.6)/ MgO(1.1)/Ta(2). The hysteresis loop shift measurement[1] is conducted to characterize the zero-field current-induced SOT effective field (H_z^{eff}/I_{DC}). Figure S1(b) shows the shifted hysteresis loops with I_{DC} = ± 6.5 mA without the application of an in-plane field. We summarize the I_{DC} dependence of switching fields and H_z^{eff} in Figure S1(c). H_z^{eff} is linearly proportional to the applied $I_{DC,}$ and the slope H_z^{eff}/I_{DC} is calculated to be 3.4 Oe/mA. This value leads to the field-free current-induced SOT switching, as shown in Figure S1(d), with a 71% switching percentage compared to the field-swept hysteresis loop. Furthermore, we perform a series of current-induced switching with different current pulse widths in Figure S1(e). In this way, the thermal stability factor can be calculated through[2]

$$J_c = J_{c0}\left[1-\frac{1}{\Delta}\ln\left(\frac{t_{pulse}}{\tau_0}\right)\right], \tag{1}$$

where J_{c0} represents the zero thermal critical switching current density, while Δ, defined as U/k_BT, is the thermal stability factor. U represents the energy barrier between two magnetization states. $1/\tau_0$ denotes the intrinsic attempt frequency, where τ_0 is approximately 1 ns[3]. Through linear fits of the data shown in Figure S1(f), J_{c0} is estimated to be 17.8 × 10^{10} A/m^2 and Δ ≈ 56.5.

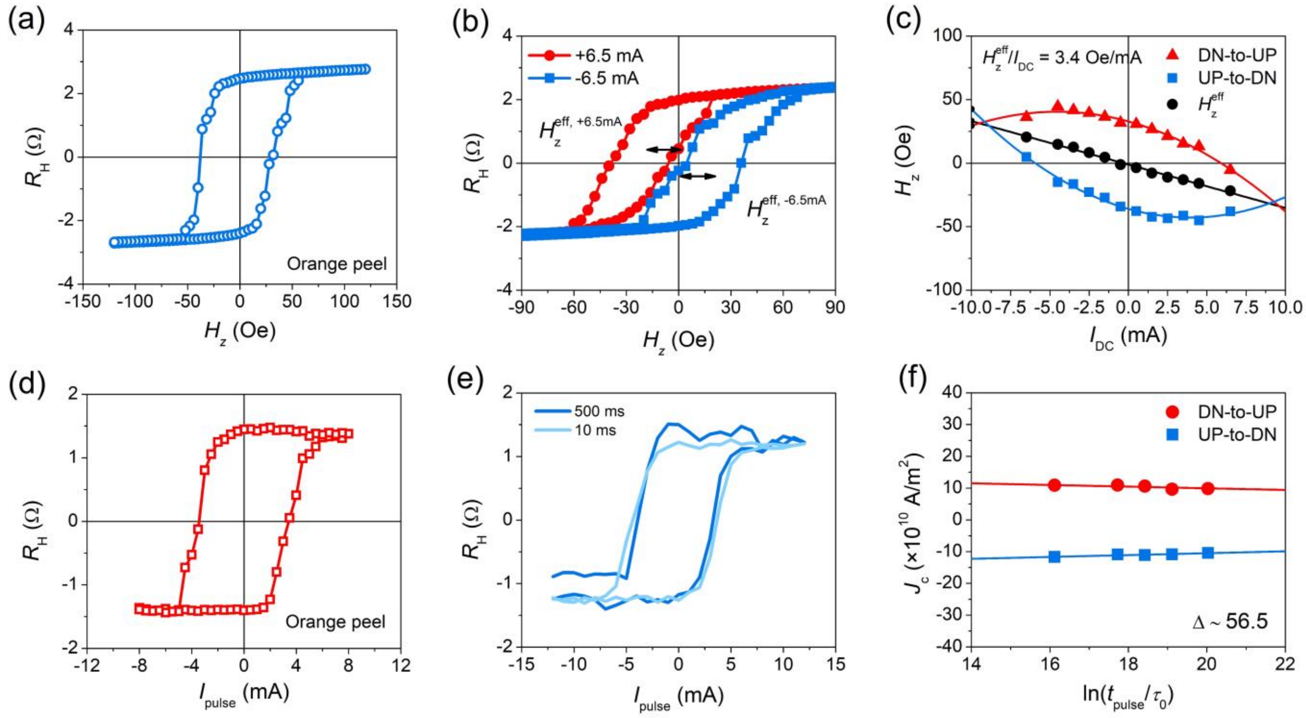

Figure S1. (a) The field-swept OOP anomalous Hall hysteresis loop. (b) Representative shifted loops under I_{DC} = ± 6.5 mA without any in-plane field. (c) The switching fields of down-to-up (red triangles), up-to-down (blue squares) transitions, and H_z^{eff} (black circles) as functions of I_{DC}. (d) Field-free current-induced magnetization switching loop. (e) Representative current-induced switching loops with various applied current pulse widths. (f) Critical switching current density, J_{c0}, as a function of the applied current pulse width.

S2. Field-free SOT switching with i-DMI

Figure S2(a) is the OOP hysteresis loop of Sample II: Ta(0.5)/CoFeB(1.2)/Pt(2.5)/Co(0.6)/Pt(0.6)/Ta(2) with i-DMI. Figure S2(b) shows the shifted hysteresis loops with I_{DC} = ± 2.5 mA without any in-plane field. We outline the I_{DC} dependence of switching fields and H_z^{eff} in Figure S2(c) and figure out that the linear slope H_z^{eff}/I_{DC} equals 9.5 Oe/mA. This large value leads to the field-free current-induced SOT switching with a 100% switching percentage, as shown in Figure S2(d). Note that we can apply the current along the -*D* direction to achieve the counterclockwise (CCW) switching loop[4-5]. Furthermore, we perform current-induced switching with different current pulse widths in Figure S2(e). In accordance with Equations (1), J_{c0} is calculated to be 20.7 × 10^{10} A/m^2 and $\Delta \approx 20.8$ from Figure S2(f).

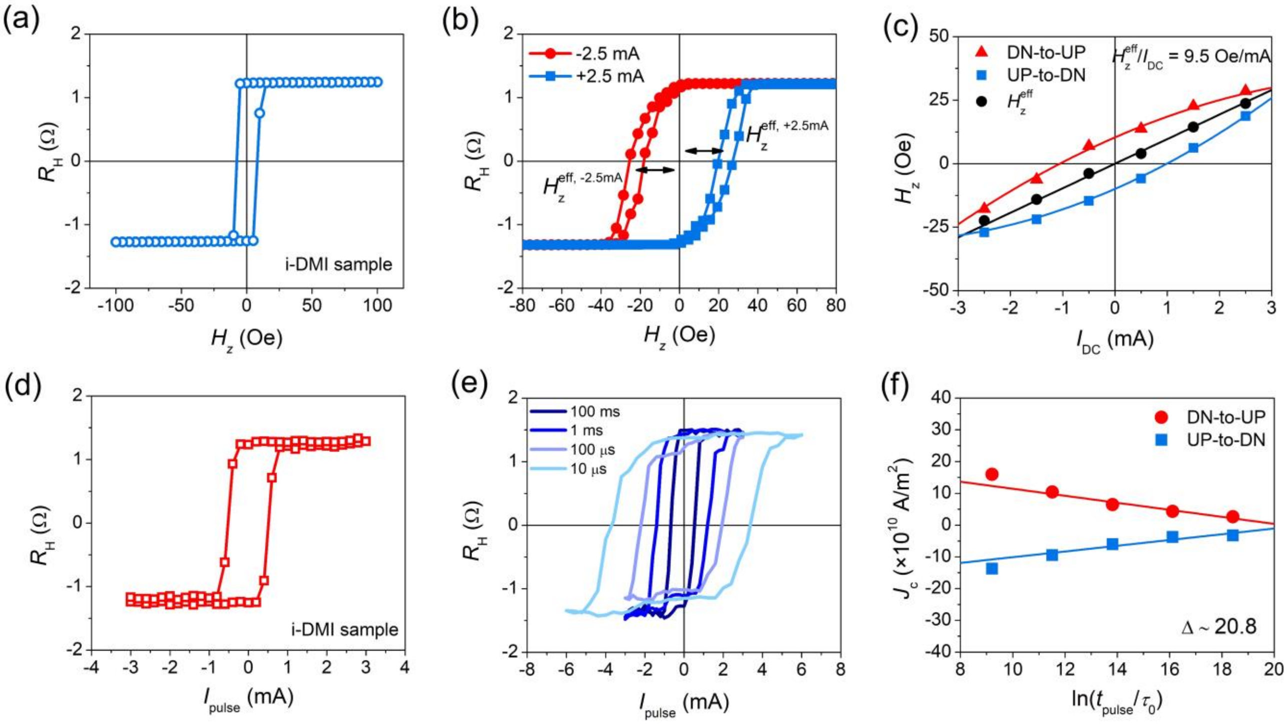

Figure S2. (a) The field-swept OOP anomalous Hall hysteresis loop. (b) Representative shifted loops under I_{DC} = ± 2.5 mA without any in-plane field. (c) The switching fields of down-to-up (red triangles),

up-to-down (blue squares) transitions, and H_z^{eff} (black circles) as functions of I_{DC}. (d) Field-free current-induced magnetization switching loop. (e) Representative current-induced switching loops with various applied current pulse widths. (f) Critical switching current density, J_{c0}, as a function of the applied current pulse width.

S3. Field-free SOT switching with tilted anisotropy

Figure S3(a) is the OOP hysteresis loop of Sample III: Ta(0.5)/Pt(6.1)/Co(0.7)/Pt(1) with tilted anisotropy. Figure S3(b) shows the shifted hysteresis loops with $I_{DC} = \pm 6.5$ mA without any in-plane field. We outline the I_{DC} dependence of switching fields and H_z^{eff} in Figure S3(c), and notably, the nonlinear trend of H_z^{eff} corresponds to the classical characteristic of tilted anisotropy[6]. This increasing H_z^{eff} with the applied current pulse also leads to a 100% field-free current-induced SOT switching, as shown in Figure S3(d). Furthermore, we perform current-induced switching with different current pulse widths in Figure S3(e). According to Equations (1), J_{c0} is calculated to be 44×10^{10} A/m^2 and $\Delta \approx 30$ from Figure S3(f). It is important to highlight that the use of a 50 μs pulse width in our micron-sized device does not indicate entry into the dynamical switching regime but is instead attributed to suboptimal electrical contact and the extended channel length in our micron-scale Hall bar geometry[4]. As a result, our study employs longer pulse durations than industry-standard nanosecond pulses typically used in nanoscale SOT devices and MTJs for memory applications[7-8].

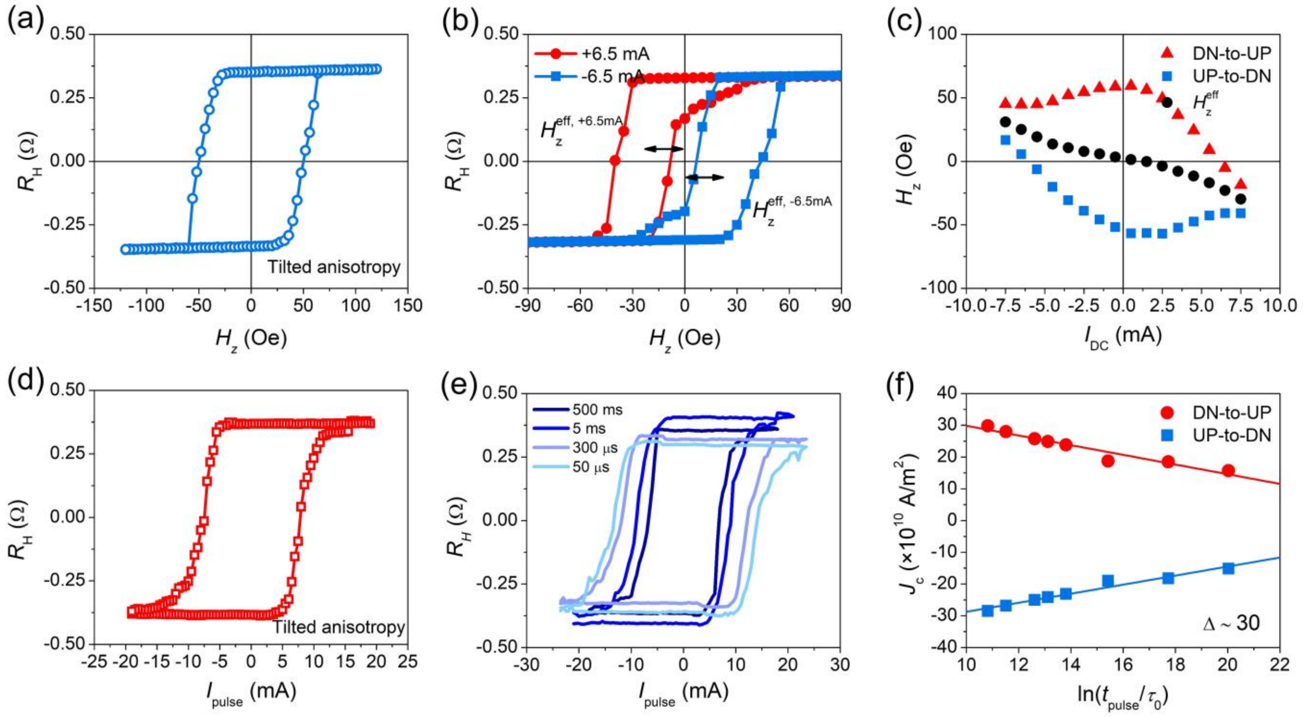

Figure S3. (a) The field-swept OOP anomalous Hall hysteresis loop. (b) Representative shifted loops under I_{DC} = ± 2.5 mA without any in-plane field. (c) The switching fields of down-to-up (red triangles), up-to-down (blue squares) transitions, and H_z^{eff} (black circles) as functions of I_{DC}. (d) Field-free current-induced magnetization switching loop. (e) Representative current-induced switching loops with various applied current pulse widths. (f) J_{c0} as a function of pulse width.

S4. Optimization of pulse width and current amplitude for multi-state switching

To systematically investigate the optimal pulse width and amplitude for maximizing the number of stable intermediate resistance states, we conducted a field-free current switching reproducibility test on the tilted anisotropy sample under varying conditions. Since both pulse width and current amplitude influence the current-induced switching behavior, identifying the optimal measurement parameters is essential for enhancing analog-like switching performance in neuromorphic computing applications.

Shorter pulse widths facilitate finer and more precise control over magnetization states, as previously discussed in Section 2.3[9]. To validate this effect, we applied a reset pulse to obtain the highest Hall resistance R_H, followed by a current pulse with a specific pulse width t_{pulse} and amplitude I_{pulse}, and subsequently measured the resulting R_H value. Each measurement was repeated 100 times to ensure statistical reliability. The results, shown in Figure S4, indicate that the tilted anisotropy sample exhibits highly stable current switching behavior across different pulse widths.

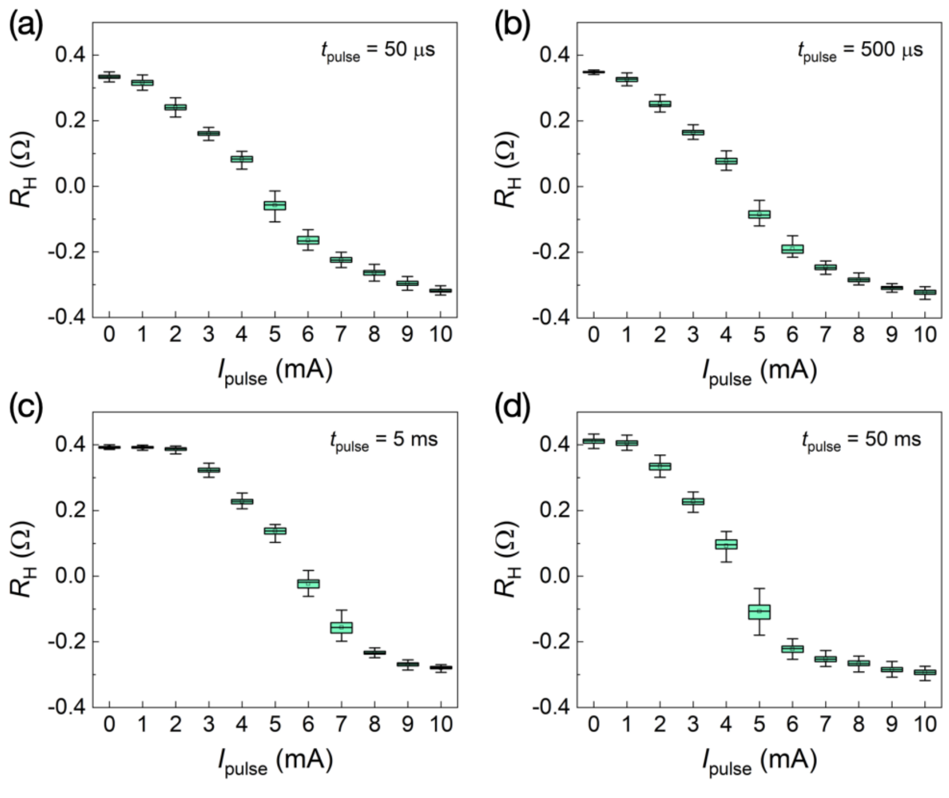

Figure S4. Reproducibility test of field-free current switching in the tilted anisotropy sample under pulse widths from (a) 50 μs, (b) 500 μs, (c) 5 ms, to (d) 50 ms. R_H values were measured after applying a reset pulse followed by specific pulse conditions, with each measurement repeated 100 times.

Further analysis of the standard deviation of R_H values for intermediate states, obtained from Figure S4, is presented in Figure S5. The results demonstrate that at t_{pulse} = 50 μs, R_H control is significantly more stable and precise. This enhanced stability may be attributed to the reduced thermal perturbation time[2, 10-11], which minimizes unwanted fluctuations during the switching process.

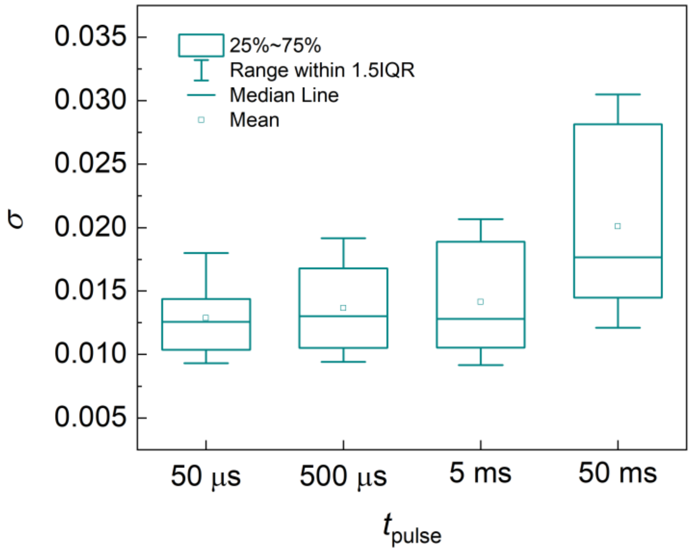

Figure S5. Standard deviation of R_H values for intermediate states under different pulse widths in the tilted anisotropy sample. Results indicate improved control and stability at shorter pulse widths.

In addition to pulse width, the amplitude of the applied current pulses also plays a crucial role in determining the number of measurable intermediate resistance states. To optimize this parameter, we fixed t_{pulse} at 50 μs and examined the analog-like switching behavior at various current amplitudes, as illustrated in Figure S6. The results indicate that when applying I_{pulse} = −13 mA, the CTC variation is minimized, and the highest number of distinguishable intermediate states is achieved. In contrast, a slightly lower I_{pulse} of -12 mA reduces the field-free switching ratio and increases CTC variation, while a slightly higher I_{pulse} of -14 mA enhances the field-free switching ratio but significantly decreases the number of distinguishable intermediate states. Similar trends were observed in the positive pulse regime, where I_{pulse} was varied between +12 and +14 mA, as shown in Figure S6(d)-(f). These observations highlight the importance of selecting an appropriate pulse amplitude to balance switching stability and the number of distinguishable states.

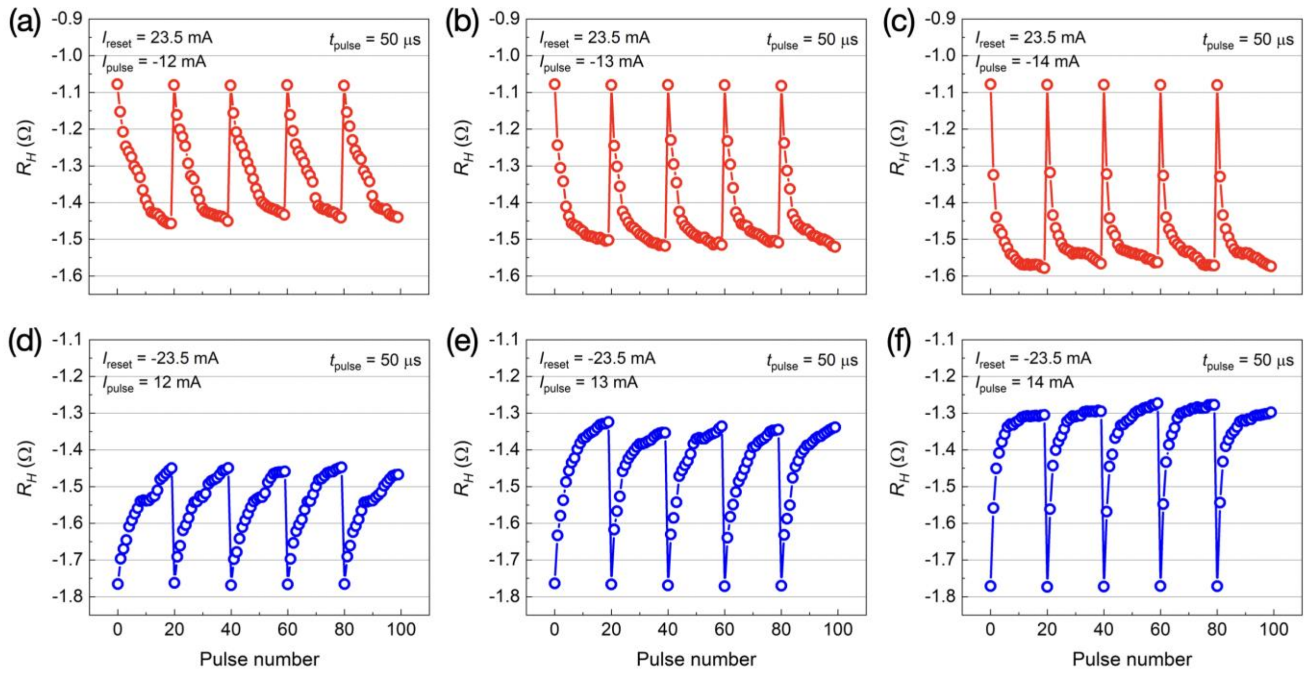

Figure S6. Analog-like switching behavior of the tilted anisotropy sample under different pulse amplitudes with a fixed pulse width of 50 μs. (a)-(c) show the results for negative pulses (-12 to -14 mA), while (d)-(f) show the results for positive pulses (+12 to +14 mA), highlighting the optimal conditions for maximizing the number of distinguishable intermediate states.

Based on these findings, the optimal measurement conditions for the tilted anisotropy sample in neuromorphic computing applications are determined to be t_{pulse} = 50 μs and I_{pulse} = ±13 mA. For the Néel orange-peel and i-DMI samples, the pulse width and amplitude were selected based on the conditions that yielded the best analog-like switching behavior. The corresponding measurement results for these samples are provided in Supporting Information Sections S5 and S6.

S5. Distinguishable states in the Néel orange-peel effect device

The same pulse number experiment is conducted on the Néel orange-peel effect device. I_{reset} and I_{pulse} are set to -6 and 3.1 mA respectively, with t_{pulse} = 50 ms, as shown in Figure S7(a). However, only 48 percent of the Hall resistance can be covered by the current pulses. We choose three states (I_{reset}, n = 1, and n = 10) to assess the CTC variation in Figure S7(c). 50 data points for each magnetic state are recorded, and the CDFs are constructed based on the measured R_H. The highest CTC variation observed here is 7.2%.

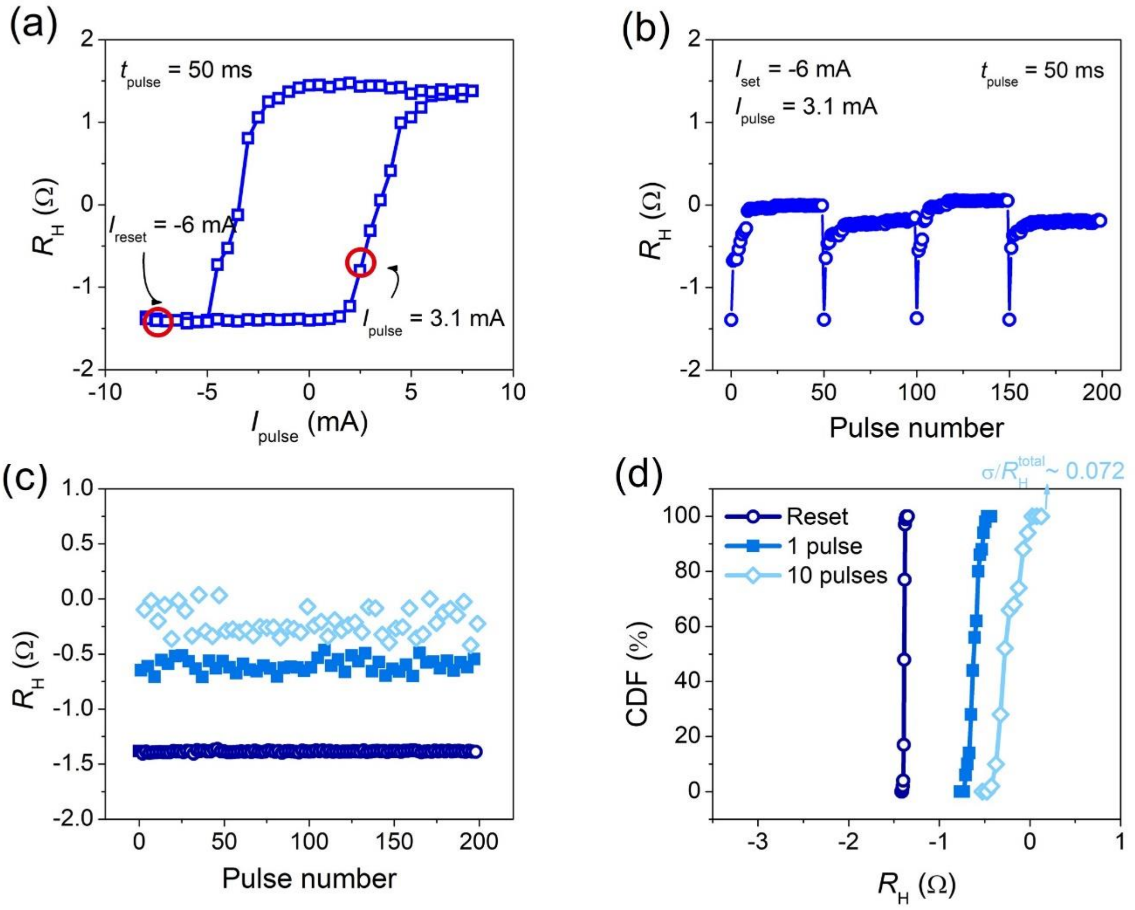

Figure S7. (a) Current-induced magnetization switching under a current pulse of 50 ms. (b) The corresponding response of R_H, where equal 49 positive current pulses of 3.1 mA are applied after a large reset current of -6 mA. (c) The response of R_H corresponds to 3 specific current pulse numbers (d) The CDFs vs. R_H for 3 distinguishable states.

S6. Distinguishable states in the i-DMI device

I_{reset} and I_{pulse} are set to -6 and 3.5 mA respectively, with t_{pulse} = 10 μs, as shown in Figure S8(a). Here, 100 % of the Hall resistance can be covered by the current pulses. We choose four states (I_{reset}, n = 1, 3, and 8) to assess the CTC variation in Figure S8(c). 50 data points for each magnetic state are recorded, and the CDFs are constructed based on the measured R_H. The highest CTC variation observed here is 4.5 %. However, the interval of the intermediate states is relatively large.

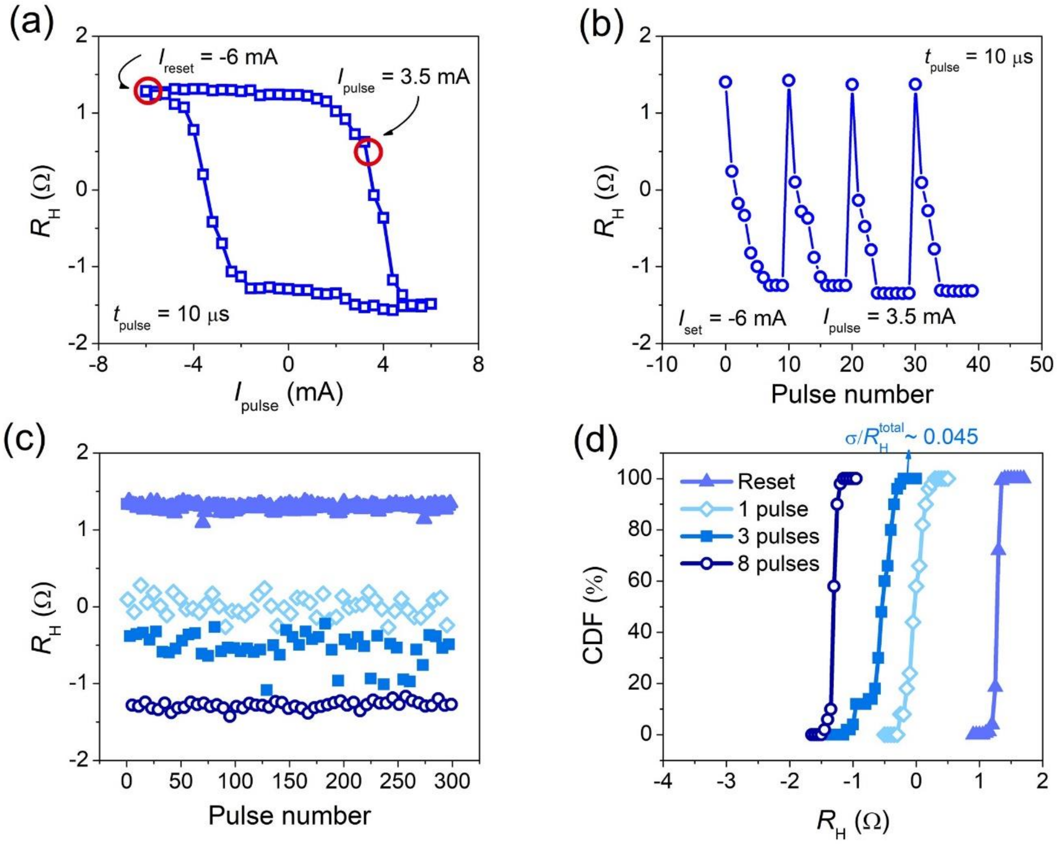

Figure S8. (a) Current-induced magnetization switching under a current pulse of 10 μs. (b) The corresponding response of R_H, where equal 9 positive current pulses of 3.5 mA are applied after a large reset current of -6 mA. (c) The response of R_H corresponds to 4 specific current pulse numbers (d) The CDFs vs. R_H for 4 distinguishable states.

S7. Assessment of device-to-device variations

In our study, performance comparisons among the Néel orange-peel, i-DMI, and tilted anisotropy devices were conducted using micron-sized Hall bar devices. This structure is known for its stable switching behavior, which effectively minimizes the impact of device-to-device variations. However, fabrication-induced inconsistencies cannot be completely excluded. To assess these variations, particularly in the tilted anisotropy sample where the oblique deposition of the bottom platinum layer during sputtering might lead to differences, current-induced magnetization switching measurements are performed on one representative sample (Sample 1) and eight neighboring devices (Samples 2–9) from the same fabrication block. The results shown in Figure S9 indicate minimal variation among these devices.

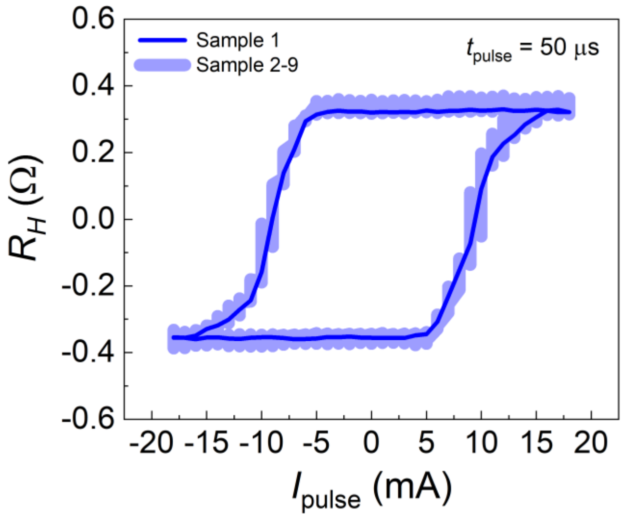

Figure S9. Current-induced magnetization switching loops of the tilted anisotropy sample, comparing the representative device (Sample 1) with its eight neighboring devices (Samples 2–9).

To further address the concern of device-to-device variations in multilevel synaptic states, we conduct additional experiments on three different tilted anisotropy devices using the same pulse amplitude and pulse width as in the main text. The cumulative distribution functions (CDFs) of the distinguishable resistance states for all three devices are shown in Figure S10. While the precise resistance values of the intermediate states differ slightly across devices, likely due to subtle variations in optimal switching conditions, each device still exhibits at least nine stable and distinguishable states. Importantly, the cycle-to-cycle variation (CTC) in these states remains below 2.7% for all devices, confirming the consistency and robustness of the switching behavior. These findings support the feasibility of implementing our tilted anisotropy devices in practical neuromorphic systems, even when accounting for device-to-device variability.

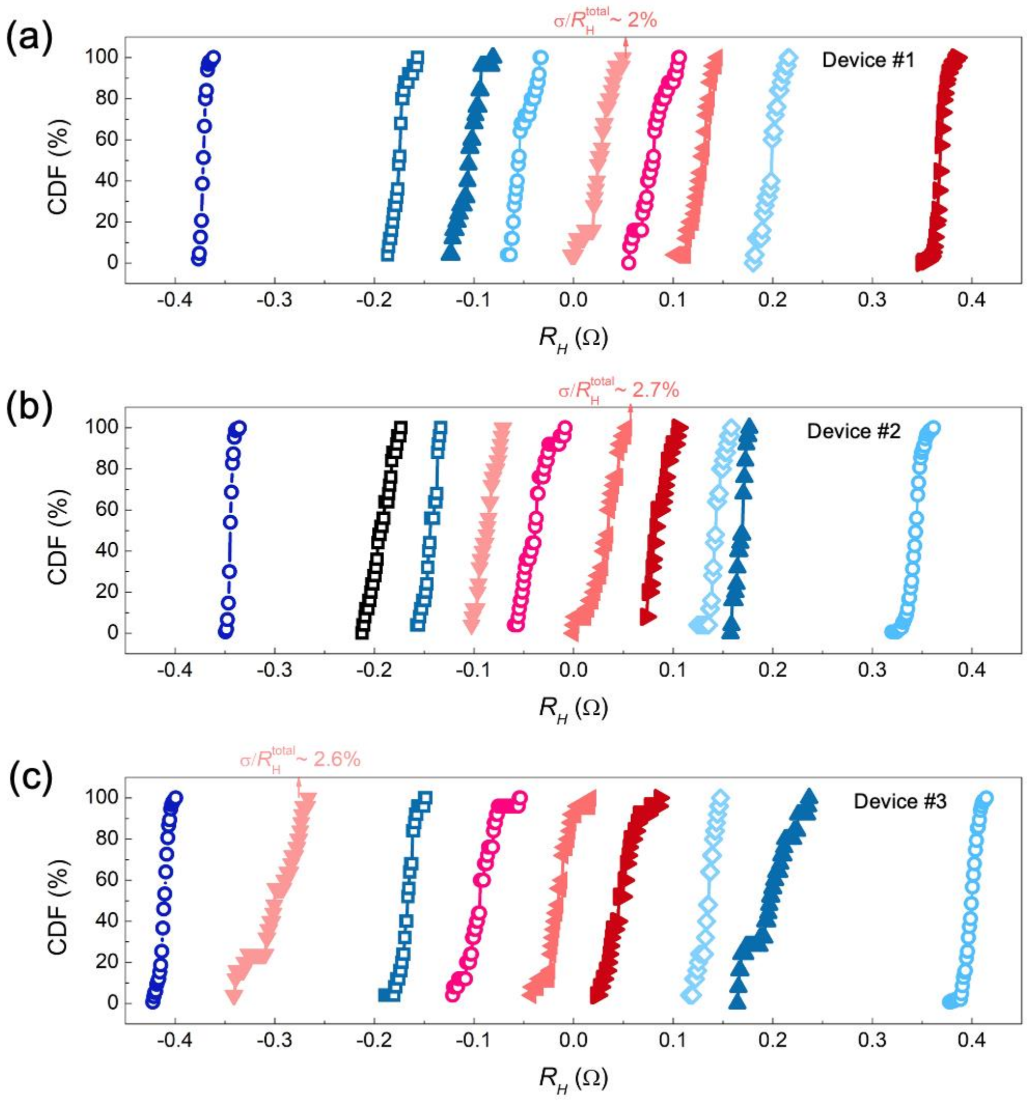

Figure S10. The cumulative distribution functions (CDFs) vs. R_H for the distinguishable states of (a) device #1, (b) device #2, and (c) device #3, all fabricated with the same tilted anisotropy structure.

S8. The characteristic resistance states ($\boldsymbol{R_{char}}$) with various pristine states

The 11 pristine Hall resistance states ($\boldsymbol{R_H}$) measured in the tilted anisotropy sample are -0.336 Ω, -0.210 Ω, -0.158 Ω, -0.110 Ω, -0.056 Ω, -0.022 Ω, 0.051 Ω, 0.097 Ω, 0.118 Ω, 0.177 Ω, 0.322 Ω. By calculating the $\boldsymbol{R_H}$ difference between pairs of memristors, the differential resistance states ($\boldsymbol{R_{diff}}$) with 110 states distributed from -0.658 Ω (-0.336-0.322) Ω) to 0.658 Ω (0.322-(-0.336) Ω) is obtained. Noted that the intervals in $\boldsymbol{R_{diff}}$ are non-uniform and so closely distributed that are prone to the inherent variability. Therefore, we further limit the difference between any two $\boldsymbol{R_{diff}}$ values to at least 2% of the total resistance (Eq. (1) in the main text) to obtain the characteristic resistance states ($\boldsymbol{R_{char}}$), and finally 36 levels of weights are derived.

For the 6 pristine states case, the $\boldsymbol{R_H}$ are -0.336 Ω, -0.210 Ω, -0.110 Ω, 0.051 Ω, 0.177 Ω, 0.322

Ω. by selecting 6 of 11 intermediate states. Following the same manner, the modified $\boldsymbol{R_{char}}$ consist of 22 levels of weights. For the 4 pristine states case, the selected $\boldsymbol{R_H}$ are -0.336 Ω, -0.110 Ω, 0.097 Ω, 0.322 Ω, and only 6 levels of weights are obtained.

S8. Quantization of weight layers in ResNet-18 using the min-max observer

The min-max observer was applied to the first convolutional layer and the fully connected (FC) layer in ResNet-18. In this process, 32-bit floating-point (FP32) weights were transformed into artificial weights using 36 weight levels. Notably, the q_{min} and q_{max} values were determined separately for the first convolutional layer and the FC layer. The distribution of artificial weights closely mirrors that of the original software weights, indicating that the FP32 weights were correctly mapped, as shown in Figure S11.

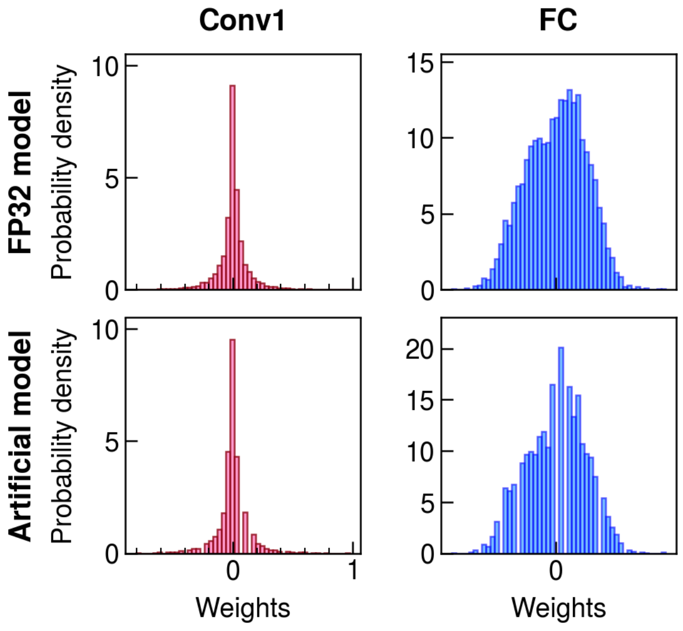

Figure S11. Distribution of artificial weights compared to software weights for the first convolutional layer and the fully connected (FC) layer in ResNet-18, using the min-max observer with 11 pristine states.

S9. The optimization of accuracy using the batch normalization (BN) observer

Figure S12 demonstrates the dependence of quantization accuracy on N across varying pristine states. For pristine states of 4, 6, and 11, the accuracy stabilizes at optimized N values of 2, 3, and 6, respectively. In this study, all quantization accuracies using the BN observer are determined based on these optimized N values.

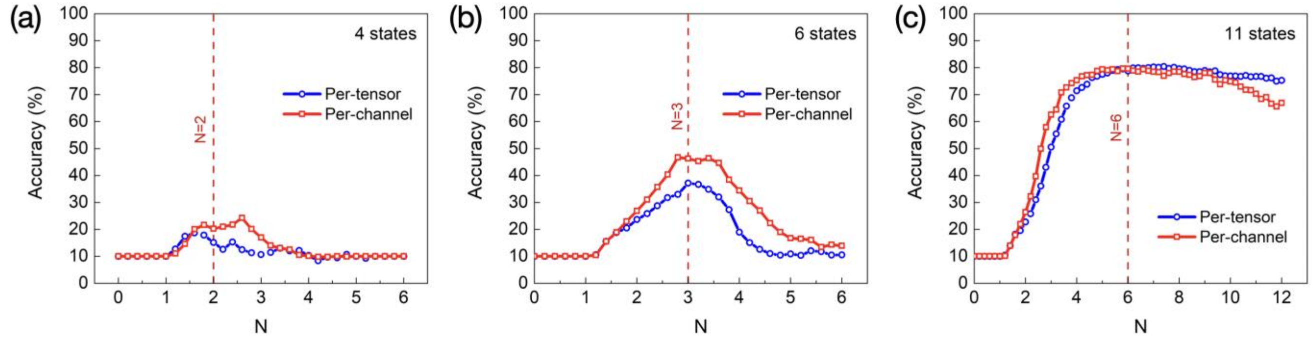

Figure S12. Dependence of quantization accuracy on N for (a) 4, (b) 6, and (c) 11 pristine states, evaluated using the BN observer.

S10. Application of post-training quantization process in a multilayer perceptron model

To further investigate the application of multi-state properties in neuromorphic computing, the 11 distinguishable states from the tilted-anisotropy sample are applied to various deep-learning models. Here, a three-layer multilayer perceptron (MLP) is constructed using the PyTorch framework, consisting of 784, 100, and 10 nodes in the input, hidden, and output layers, respectively, as illustrated in Figure S13a. The activation function used is Sigmoid, and the model is trained on the MNIST dataset, which includes 40,000 training images, 10,000 validation images, and 10,000 testing images, each with dimensions of 28×28 pixels. The training is performed with a mini-batch size of 10, a learning rate of 0.005, and over 20 epochs, achieving training and testing accuracies of 98.41% and 97.55%, respectively.

The quantization results for the two fully connected layers (FC1 and FC2) in the MLP model, where 32-bit floating-point (FP32) weights are mapped to artificial weights using the min-max observer with 36 quantization levels, are shown in Figure S13b. The q_{min} and q_{max} values for FC1 and FC2 are determined separately. The artificial weight distribution closely aligns with the software weight distribution, indicating that FP32 weights are accurately mapped.

The classification accuracy of the quantized MLP model, with and without an observer, for pristine states of 4, 6, and 11, is presented in Figure S13c. After applying observers—including min-max, MSE, and BN observers—accuracy generally improves, except in the case of 4 pristine states quantized with the MSE observer, which exhibits a notable drop. However, performance significantly improves when the state number increases to 6, highlighting the MSE observer's sensitivity to large outliers and the necessity of a sufficiently high state number. With 11 pristine states, the overall quantization accuracy approaches the baseline performance, demonstrating the importance of a higher number of artificial weight levels for optimal performance in hardware-implemented neural networks.

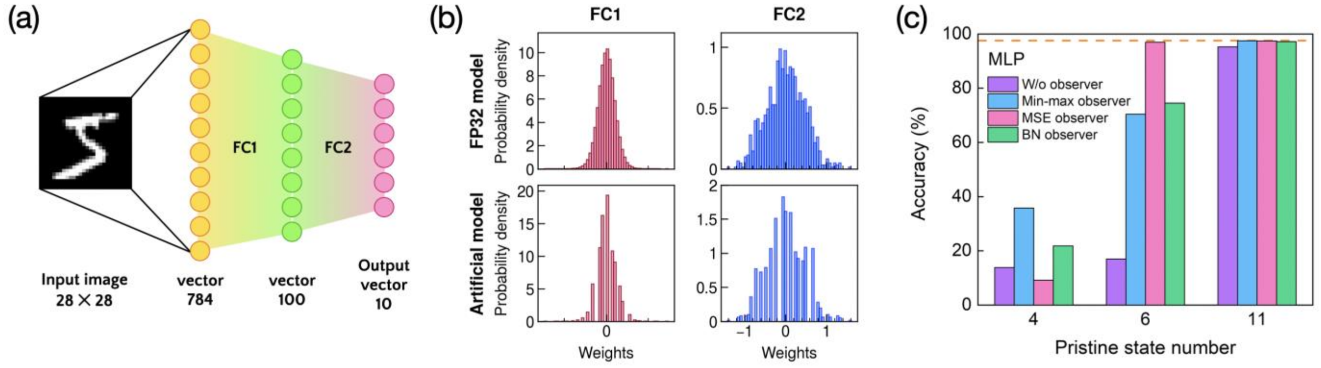

Figure S13. Architecture of the three-layer MLP, consisting of 784, 100, and 10 nodes in the input, hidden, and output layers, respectively. (b) Distribution of artificial weights compared to software weights for the fully connected layers (FC1 and FC2) in the MLP model, obtained using the min-max observer with 36 weight levels. (c) Classification accuracy of the quantized MLP model for pristine states of 4, 6, and 11, with and without an observer. The dashed line represents the baseline accuracy of 97.55%.

S11. Weight statistics for per-tensor quantization

In Figure S14, the weight statistics for the first convolutional layer (32×7×7×3) are presented both before quantization (FP32) and after quantization using various observers (min-max, MSE, BN) with 36 levels of weights (i.e., 11 pristine states). Specifically, the min-max observer and the MSE method yield maximum and minimum values that are close to those of the FP32 model, indicating a susceptibility to outliers. In contrast, the BN observer exhibits markedly different maximum and minimum values due to the selective determination of q_{min} and q_{max}; however, its overall weight distribution, including the 1.5 interquartile range (IQR), closely mimics that of the FP32 model.

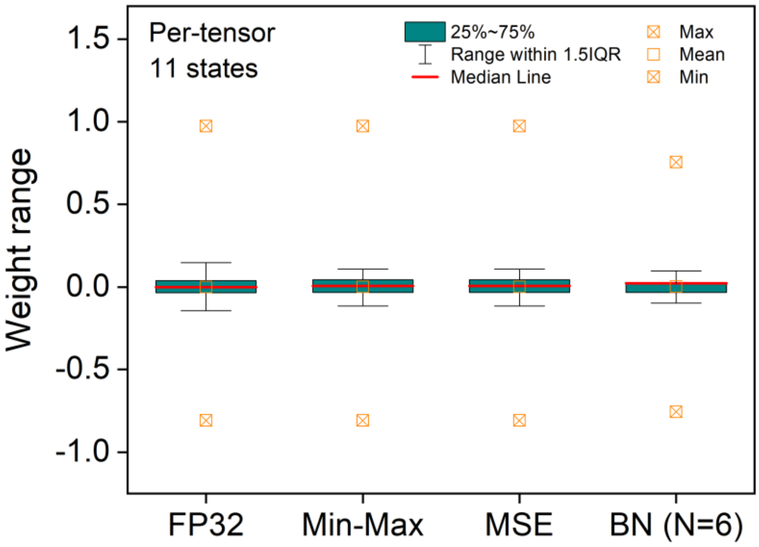

Figure S14. Weight statistics of the first convolutional layer for the FP32 ResNet-18 model, as well as per-tensor quantization using min-max, MSE, and BN observers.

S12. Weight statistics for per-channel quantization

To quantitatively assess the relationship between quantization performance and accuracy in per-channel quantization, we calculated the mean squared error before and after quantization. Taking the first convolutional layer as an example, Figure S15 presents the distribution of weight transfer error across its 64 output channels. The analysis reveals that the MSE observer exhibits the lowest error during the weight transfer process, while the min-max observer shows a similarly low yet slightly higher error. In contrast, the BN observer records the highest MSE value. This elevated error for the BN observer may explain its lower quantization accuracy (79.58%) observed in the 11 pristine state case, as further indicated by the corresponding data in Table S1.

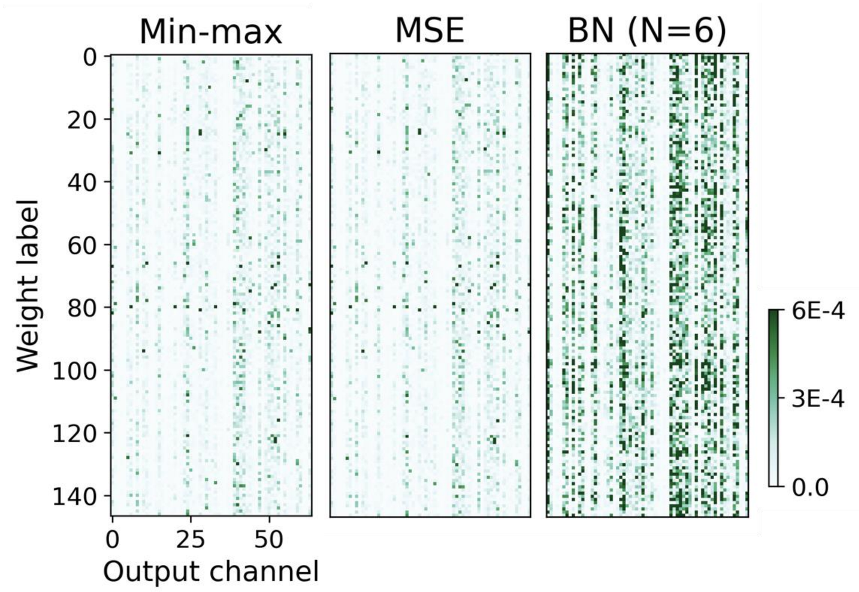

Figure S15. Weight transfer error across the 64 output channels of the first convolutional layer for different observers.

S13. Comparison of classification accuracy for three quantization schemes in CNN model

Table S1. Classification accuracy (%) of the per-tensor and per-channel quantized ResNet-18 model at pristine states of 4, 6, and 11 with and without using the observer.

Quantization scheme	Type of observer	Pristine state number		
		4	6	11
	N/A	10.00	10.00	45.88
Per-tensor	Min-max observer	10.00	10.00	79.22
	MSE observer	10.00	10.00	79.12
	BN observer	18.02	37.13	78.77
Per-channel	Min-max observer	10.00	10.59	81.51
	MSE observer	10.00	13.06	81.12
	BN observer	22.57	46.27	79.58

REFERENCES

[1] C.-F. Pai, M. Mann, A. J. Tan, G. S. Beach. *Phys. Rev. B* **2016**, 93, 144409.

[2] R. Koch, J. Katine, J. Sun. *Phys. Rev. Lett.* **2004**, 92, 088302.

[3] A. Khvalkovskiy, D. Apalkov, S. Watts, R. Chepulskii, R. Beach, A. Ong, X. Tang, A. Driskill-Smith, W. Butler, P. Visscher. *J. Phys. D: Appl. Phys.* **2013**, 46, 074001.

[4] C.-Y. Lin, P.-C. Wang, Y.-H. Huang, W.-B. Liao, M.-Y. Song, X. Bao, C.-F. Pai. *ACS Mater. Lett.* **2023**, 6, 400.

[5] C.-Y. Lin, J.-Y. Hsieh, P.-C. Wang, C.-C. Tsai, C.-F. Pai. *APL Mach. Learn.* **2024**, 2, 046110.

[6] C.-Y. Hu, W.-D. Chen, Y.-T. Liu, C.-C. Huang, C.-F. Pai. *NPG Asia Mater.* **2024**, 16, 1.

[7] M. Song, C. Lee, S. Yang, G. Chen, K. Chen, I. J. Wang, Y. Hsin, K. Chang, C. Hsu, S. Li, presented at *2022 IEEE Symposium on VLSI Technology and Circuits (VLSI Technology and Circuits),* **2022**.

[8] K. Cai, S. Van Beek, S. Rao, K. Fan, M. Gupta, V. Nguyen, G. Jayakumar, G. Talmelli, S. Couet, G. S. Kar, presented at *2022 IEEE Symposium on VLSI Technology and Circuits (VLSI Technology and Circuits),* **2022**.

[9] A. Kurenkov, S. DuttaGupta, C. Zhang, S. Fukami, Y. Horio, H. Ohno. *Adv. Mater.* **2019**, 31, 1900636.

[10] W. F. Brown Jr. *Phys. Rev.* **1963**, 130, 1677.

[11] D. C. Ralph, M. D. Stiles. *J. Magn. Magn. Mater.* **2008**, 320, 1190.
